# Supplementary material for: STOP-Colitis pilot trial protocol: a prospective, open-label, randomised pilot study to assess two possible routes of faecal microbiota transplant delivery in patients with ulcerative colitis
Source: BMJ Open. 2019 Nov 11;9(11):e030659. doi: 10.1136/bmjopen-2019-030659 (PMC6858155; doi:10.1136/bmjopen-2019-030659)
Supplement: Supplementary data [file bmjopen-2019-030659supp001.pdf]

INTERVIEW SCHEDULE STOP-COLITIS T1 (Baseline) - PATIENTS

INTRODUCTION

Thank the participant for agreeing to take part.

Ensure that the consent form has been signed. Keep a copy for the site file and give a copy back to the participant.

Statement on confidentiality, right to withdraw consent, recording of the interview.

Review the purpose of the study in general.

Emphasize the value of their views and opinions – there are no right or wrong answers.

Ask if the participant have any questions before starting the interview.

ICEBREAKER

Background, interviewee details and icebreaker e.g. explore interviewee background, have family, and why they were interested in taking part in this particular interview/research etc.

BACKGROUND INFORMATION (provides context for remainder of data) – Interviewees' experience of UC (symptoms, diagnosis, treatment, impact)

Brief discussion of period leading to diagnosis (e.g. when, symptoms, impact, presentation, initial treatment, knowledge of UC, reaction to diagnosis)

Brief discussion of experience post diagnosis (e.g. experience of flare and remission, treatment, impact, adaptations)

PERSPECTIVES ON TRIAL RECRUITMENT, PARTICIPATION AND PROCEDURES

1. What are your reasons/motivations for taking part in STOP-COLITIS?
    - How long did it take you to decide to take part?
    - Did you make the decision on your own or did you ask other people's opinion to help you decide? If so, who? And why?
2. Patients' understanding of STOP-COLITIS
    - In your own words, what do you think the aims of STOP-COLITIS are?
    - What were you told about it?
3. Patients' understanding/expectations of FMT
    - Were you aware of FMT before being introduced to STOP-COLITIS?

- What is your understanding of FMT? NG? Colon?
- What were you told about FMT before consenting to the trial?
- How do you feel about receiving someone else's stool sample?
- How do you think it compares with other treatments (medical treatment)?
- Do you know what the two groups (NG/Colon) entail?
- Do you have any preferences as to which group you would like to be randomised to?
- Have you talked with other people about the trial or FMT? Have you told your partner/close family about what FMT is?
- How would you react to being randomised the NG group? The Colonoscopy group?
- What do your close family and partner/parents think about FMT?

4. Expectations for trial participation
   - What do you expect from taking part in STOP-COLITIS?
5. Experience of information and consent received from medical team
   - Type of information received
   - Who provided the information?
   - Was it useful?
   - Enough time to digest it? To think about the study?
   - What other information would patients have liked (if any)?
6. Potential barriers/facilitators to patients' participation in STOP-COLITIS
   - What might influence patients to participate?
   - Why might patients decide not to take part in STOP-COLITIS?
   - What could be done to encourage them taking part in STOP-COLITIS?
7. Experience of different stages of trial
   - How would you describe your experience of consenting to STOP-COLITIS?
   - Who took consent?
   - Did you have enough time to think about it before consenting?
   - Do you know what "random allocation" is in STOP-COLITIS and what it involves? Who did the randomisation?
   - Do you know what the follow-up process entails in STOP-COLITIS? Questionnaires/Blood tests/stool samples etc…?
8. Outcomes
   - What should we be comparing/measuring in these questionnaires etc…?
   - What is important to you (in terms of symptoms, treatment, QoL, etc…)

9. The main trial, after this pilot trial, will include a placebo group and an intervention group (NG or colon?), hypothetically, Would you have felt differently about STOP-COLITIS if there was a possibility that you might get the placebo rather than the FMT?
10. I have come to the end of my questions, do you have any other issues you wish to talk about?

CLOSING

Tell the participants that they have reached the end of the interview

Check understanding of any outstanding points and give the participant the opportunity to answer ask any further questions.

Check arrangements for scheduling the follow up interview

Remind them about confidentiality and thank them for their time

INTERVIEW SCHEDULE STOP-COLITIS T2 (12 weeks) - PATIENTS

INTRODUCTION

Thank the participant for agreeing to take part.

Ensure that the consent form has been signed. Keep a copy for the site file and give a copy back to the participant.

Statement on confidentiality, right to withdraw consent, recording of the interview.

Review the purpose of the study in general.

Emphasize the value of their views and opinions – there are no right or wrong answers.

Ask if the participant has any questions before starting the interview.

HOW PATIENTS FEEL AFTER 12 WEEKS MONTHS

1. How have you been in general since the last time I saw you?
2. What symptoms have you experienced over the last 3 months? (any changes since initial interview?)
3. Have you had any flare ups since initial interview? Types/Severity/Frequency of symptoms? Treatment?
4. Has anything changed since the last time I saw you?

PILOT TRIAL PROCESSES AND PROCEDURES

1. How did you feel about being randomised to NG/Colon?
    - Were you hoping to be randomised to NG/Colon?
2. What was your experience of FMT (NG)?
    - Colonoscopy?
    - Medication prior to/after FMT infusion?
    - Intubation?
    - Attending the CRF daily?
    - Did it match your expectations?
    - Do you wish you had been randomised to the other group? Why? Why not?
3. What was your experience of FMT (Colon) and recovery?
    - Colonoscopy?
    - Medication prior to/after FMT dose?
    - Endoscopy?
    - Enema?

- Attending the CRF weekly?

4. What was your experience of the clinical reviews/assessments after the intervention?
   - What did you think of the QoL questionnaires you had to complete?
   - What did you think of IBD diaries? Did you complete them? How did you find it?
   - What did you think of the stool kit? Was it easy to use?
   - Is there anything that could have been done differently as far as these follow-up assessments are concerned?
5. Overall, have your initial expectations of taking part in the trial been met? Have these expectations changed?
6. What do you think are the barriers and facilitators to patients' participation in STOP-COLITIS?
7. Is there anything that you think we should do differently?

CLOSING

Tell the participants that they have reached the end of the interview.

Check understanding of any outstanding points and give the participant the opportunity to answer ask any further questions.

Remind them about confidentiality and thank them for their time.

INTERVIEW SCHEDULE STOP-COLITIS – WITHDRAWAL PATIENTS

INTRODUCTION

Thank the participant for agreeing to take part

Ensure that the consent form has been signed. Keep a copy for the site file and give a copy back to the participant

Statement on confidentiality, right to withdraw consent, recording of the interview

Review the purpose of the study in general

Emphasize the value of their views and opinions – there are no right or wrong answers

Ask if the participant have any questions before starting the interview

BACKGROUND/ICE BREAKER

What are your main reasons for agreeing to talk to me (qual researcher)?

HOW PATIENTS FEEL AFTER 3 MONTHS

5. How have you been in general since the last time I saw you?
6. What symptoms have you experienced over the last 3 months? (any changes since initial interview?)
7. Have you had any flare ups since initial interview? Types/Severity/Frequency of symptoms? Treatment?
8. Has anything changed since the last time I saw you?

PILOT TRIAL PARTICIPATION AND PROCEDURES

8. How did you feel about being randomised to NG/Colon?
   - Were you hoping to be randomised to NG/Colon?
9. What was your experience of FMT (NG)?
   - Colonoscopy?
   - Medication prior to/after FMT infusion?
   - Intubation?
   - Attending the CRF daily?
   - Did it match your expectations?
   - Do you wish you had been randomised to the other group? Why? Why not?
10. What was your experience of FMT (Colon)?
    - Colonoscopy?

- Medication prior to/after FMT dose?
- Endoscopy?
- Enema?
- Attending the CRF weekly?

11. What was your experience of the clinical reviews/assessments after the intervention? *(if applicable)*
    - What did you think of the QoL questionnaires you had to complete?
    - What did you think of IBD diaries? Did you complete them? How did you find it?
    - What did you think of the stool kit? Was it easy to use?
    - What do you we should have done differently as far as these follow-up assessments are concerned?
12. Overall, have your initial expectations of taking part in the trial been met? Have these expectations changed?

REASONS FOR WITHDRAWING FROM STOP-COLITIS

13. What were your main reasons for withdrawing from the trial?
    - Did you take the decision on your own? Did you ask for other people's advice? If so, who?
14. What else would make patients decide to withdraw from the study?
15. What could be done to prevent patients from withdrawing from the study?
16. What do you think we should do differently overall?

CLOSING

Tell the participants that they have reached the end of the interview

Check understanding of any outstanding points and give the participant the opportunity to answer ask any further questions.

Remind them about confidentiality and thank them for their time

INTERVIEW SCHEDULE STOP-COLITIS – PATIENT DECLINERS

INTRODUCTION

Thank the participant for agreeing to take part

Ensure that the consent form has been signed. Keep a copy for the site file and give a copy back to the participant

Statement on confidentiality, right to withdraw consent, recording of the interview

Review the purpose of the study in general

Emphasize the value of their views and opinions – there are no right or wrong answers

Ask if the participant have any questions before starting the interview

BACKGROUND/ICEBREAKER

Background, interviewee details and icebreaker e.g. explore interviewee background, whether they have family, etc…

BACKGROUND INFORMATION (provides context for remainder of data) – INTERVIEWEES' EXPERIENCE TO DATE WITH UC (symptoms, diagnosis, treatment, impact)

Brief discussion of period leading to diagnosis (e.g. when, symptoms, impact, presentation, initial treatment, knowledge of UC, reaction to diagnosis)

Brief discussion of experience post diagnosis (e.g. experience of flare and remission, treatment, impact, adaptations)

PERSPECTIVES ON TRIAL RECRUITMENT AND PARTICIPATION

11. Patients' understanding of STOP-COLITIS
    - In your own words, what do you think the aims of STOP-COLITIS are?
    - Do you know what "random allocation" is in STOP-COLITIS and what it involves?
12. Patients' understanding/expectations of FMT
    - Were you aware of FMT before being introduced to STOP-COLITIS?
    - What is your understanding of FMT? NG? Colon?
    - Do you know what the two groups (NG/Colon) entail?
13. Experience of information received from medical team
    - Type of information received

- Who provided the information?
- Was it useful?
- Would patients have liked other information? If so, what information?

REASONS FOR DECLINING TO PARTICIPATE

14. What are your reasons/motivations for not taking part in STOP-COLITIS?
    - How long did it take you to decide not to take part?
    - Did you make the decision on your own or did you ask other people's opinion to help you decide? If so, who? And why?
15. Potential barriers/facilitators to STOP-COLITIS
    - What might influence patients to participate?
    - What else might make patients decide not to take part in STOP-COLITIS?
    - What could be done to encourage them taking part in STOP-COLITIS?
16. What do you think we could do differently overall?
17. Do you have any other issues you wish to talk about?

CLOSING

Tell the participants that they have reached the end of the interview

Check understanding of any outstanding points and give the participant the opportunity to answer ask any further questions.

Remind them about confidentiality and thank them for their time
