# Supplementary figures and images for: STOP-Colitis pilot trial protocol: a prospective, open-label, randomised pilot study to assess two possible routes of faecal microbiota transplant delivery in patients with ulcerative colitis
Source: BMJ Open. 2019 Nov 11;9(11):e030659. doi: 10.1136/bmjopen-2019-030659 (PMC6858155; doi:10.1136/bmjopen-2019-030659)

Supplementary Figure 1 – Trial Schema

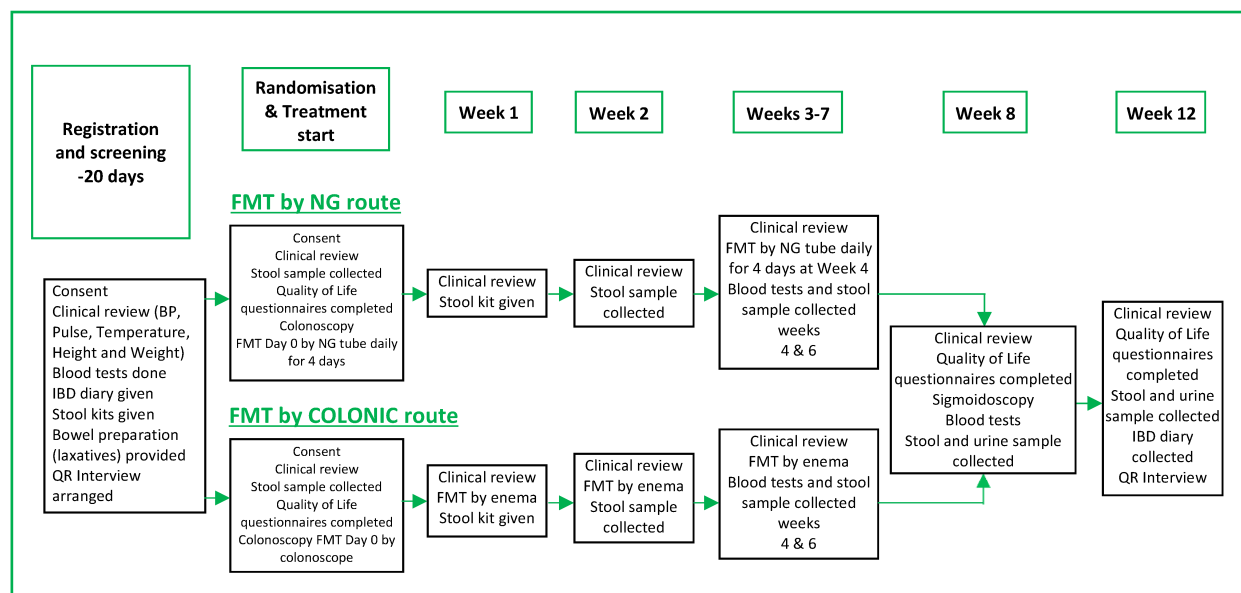

Supplement: Supplementary data [file bmjopen-2019-030659supp002.pdf]
