# Supplementary material for: STOP-Colitis pilot trial protocol: a prospective, open-label, randomised pilot study to assess two possible routes of faecal microbiota transplant delivery in patients with ulcerative colitis
Source: BMJ Open. 2019 Nov 11;9(11):e030659. doi: 10.1136/bmjopen-2019-030659 (PMC6858155; doi:10.1136/bmjopen-2019-030659)
Supplement: Author's manuscript [file bmjopen-2019-030659.draft_revisions.pdf]

BMJ Open is committed to open peer review. As part of this commitment we make the peer review history of every article we publish publicly available.

When an article is published we post the peer reviewers' comments and the authors' responses online. We also post the versions of the paper that were used during peer review. These are the versions that the peer review comments apply to.

The versions of the paper that follow are the versions that were submitted during the peer review process. They are not the versions of record or the final published versions. They should not be cited or distributed as the published version of this manuscript.

BMJ Open is an open access journal and the full, final, typeset and author-corrected version of record of the manuscript is available on our site with no access controls, subscription charges or pay-per-view fees (http://bmjopen.bmj.com).

If you have any questions on BMJ Open's open peer review process please email info.bmjopen@bmj.com

BMJ Open

Title: Stop-Colitis pilot trial protocol. A prospective, open-label, randomised pilot study to assess two possible routes of Faecal Microbiota Transplant delivery in patients with ulcerative colitis

Journal:	*BMJ Open*
Manuscript ID	bmjopen-2019-030659
Article Type:	Protocol
Date Submitted by the Author:	28-Mar-2019

Complete List of Authors:	Quraishi, Mohammed Nabil; University of Birmingham, University of Birmingham Microbiome Treatment Centre; University of Birmingham<br>Yalchin, Mehmet; St Marks Hospital<br>Blackwell, Clare; Glasgow Royal Infirmary<br>Segal, Jonathan; St Marks Hospital<br>Sharma, Naveen; Heart of England NHS Foundation Trust, Department of Gastroenterology<br>Hawkey, Peter; University of Birmingham, Institute of Microbiology and Infection<br>McCune, Victoria; University of Birmingham, Institute of Microbiology and Infection; Public Health England Midlands and East Region, Public Health Laboratory Birmingham<br>Hart, Ailsa L.; St Marks Hospital<br>Gaya, Daniel; Glasgow Royal Infirmary<br>Ives, Natalie; University of Birmingham, Clinical Trials Unit<br>Magill, Laura ; University of Birmingham, Clinical Trials Unit<br>Loi, Shrushma; University of Birmingham, Clinical Trials Unit<br>Hewitt, Catherine; University of Birmingham, Clinical Trials Unit<br>Gerasimidis, Konstantinos; University of Glasgow, Human Nutrition<br>Loman, Nicholas; University of Birmingham, Institute of Microbiology and Infection<br>Hansen, Richard; Royal Hospital for Children Glasgow, Department of Paediatric Gastroenterology<br>McMullan, Christel; University of Birmingham, Institute of Applied Health Research<br>Mathers, Jonathan; University of Birmingham, Public Health<br>Quince, Christopher; University of Warwick Warwick Medical School<br>Crees, Nicola; Crohn's and Colitis UK<br>Iqbal, Tariq; University of Birmingham, University of Birmingham Microbiome Treatment Centre; Queen Elizabeth Hospital

Keywords:	Inflammatory bowel disease < GASTROENTEROLOGY, MICROBIOLOGY, Clinical trials < THERAPEUTICS

Title: Stop-Colitis pilot trial protocol. A prospective, open-label, randomised pilot study to assess two possible routes of Faecal Microbiota Transplant delivery in patients with ulcerative colitis

Authors:

Mohammed Nabil Quraishi[1,2], Mehmet Yalchin[3], Clare Blackwell[4], Jonathan Segal[3], Naveen Sharma[5], Peter Hawkey[6], Victoria L McCune[6,7], Ailsa Hart[3], Daniel R Gaya[4], Natalie Ives[8], Laura Magill[8], Shrushma Loi[8], Catherine A Hewitt[8], Konstantinos Gerasimidis[9], Nicholas Loman[6], Richard Hansen[10], Christel McMullan[11], Jonathan Mathers[11], Chris Quince[12], Nicola Crees[13], Tariq Iqbal[1,2]

Affiliations:

[1]University of Birmingham Microbiome Treatment Centre, University of Birmingham, Birmingham

[2]Department of Gastroenterology, University Hospital Birmingham, Birmingham

[3]St Marks Hospital, London

[4]Gastroenterology Unit, Glasgow Royal Infirmary, Glasgow

[5]Department of Gastroenterology, Heart of England Foundation Trust, Birmingham

[6]Institute of Microbiology and Infection, University of Birmingham, Birmingham

[7]Public Health England, Public Health Laboratory Birmingham, Birmingham, United Kingdom

[8]Birmingham Clinical Trials Unit, Institute of Applied Health Research, University of Birmingham, Birmingham

[9]Human Nutrition, School of Medicine, Dentistry and Nursing, Glasgow Royal Infirmary, University of Glasgow, Glasgow

[10]Department of Paediatric Gastroenterology, Royal Hospital for Children, Glasgow

[11]Institute of Applied Health Research, University of Birmingham, Birmingham

[12]Warwick Medical School, Microbiology and Infection, University of Warwick

[13]Crohns and Colitis UK, United Kingdom

Corresponding author: Professor Tariq Iqbal; t.h.iqbal@bham.ac.uk

For peer review only - http://bmjopen.bmj.com/site/about/guidelines.xhtml

ABSTRACT

Introduction

Imbalance of the gut microbiome is key to the pathogenesis of ulcerative colitis (UC). Faecal microbiota transplant (FMT) is the transfer of homogenised and filtered faeces from a healthy individual to the gastrointestinal tract of a patient with disease. Published datasets show a positive signal for the use of FMT to treat UC, but the optimal route and dose of FMT remains unanswered.

Methods and analysis

This prospective, multi-centre open-label, randomised pilot study will assess two possible routes of FMT delivery, via the naso-gastric route or by delivery to the colon, in 30 patients with active UC recruited from three sites in the UK. Stool will be collected from healthy screened donors, processed, frozen and stored under an MHRA manufacturing licence held at the University of Birmingham Microbiome Treatment Centre. Thawed FMT samples will be administered to patients either via 8 naso-gastric infusions given over 4 days starting on the day of randomisation, and then again for 4 days in week 4 for foregut delivery (total of 240g of stool) or via one colonoscopic infusion followed by 7 weekly enemas according to the hindgut protocol (total of 360g of stool). Patients will be followed up weekly for 8 weeks, and then at 12 weeks. The aims of this pilot study are i) to determine which FMT administration route (naso-gastric or colon) should be investigated in a randomised double-blind, placebo-controlled trial; and ii) to determine if a full randomised controlled trial is feasible. The primary outcome will be a composite assessment of both qualitative and quantitative data based on efficacy (clinical response), acceptability and safety. At the end of the pilot, decisions will be made regarding the feasibility of a full randomised double-blind, placebo-controlled trial and, if deemed feasible, which route of administration should be used in such a study.

For peer review only

Strengths and limitations of this study

<u>Strengths</u>

- Matching of single donor with each patient;
- Intensive FMT treatment regimen;
- Assessment of mechanistic outcomes and donor diet;
- Qualitative data from clinicians and patients informing the potential future randomised controlled trial.

<u>Limitations</u>

For peer review only - http://bmjopen.bmj.com/site/about/guidelines.xhtml

- This study is limited to assessing the feasibility of a randomised controlled trial and the best route of FMT delivery in UC patients. The assessment of the efficacy of FMT in achieving and maintaining remission for patients with UC needs to be tested in a future full-scale randomised controlled trial.

Ethics and dissemination

Ethical approved for this study has been obtained from the East Midlands-Nottingham Research Ethics Committee (REC 17/EM/0274). At the end of the study, findings will be reported at national and international gastroenterology meetings and published in peer-reviewed journals.

Trial registration number ISRCTN74072945

Registered with: International Standard Registered Clinical Study Number (ISRCTN).

Data set: 74072945

Protocol version: 4.0 17-Aug-2018

Funder: Efficacy and Mechanism Evaluation - NIHR

Sponsor: University of Birmingham

Keywords: Faecal Microbiota Transplant, Ulcerative Colitis, Randomised Controlled Pilot Study

Word Count of manuscript: 5185

For peer review only

INTRODUCTION

In the inflammatory bowel diseases (IBD), Crohn's disease (CD) and ulcerative colitis (UC), there is imbalance or 'dysbiosis' of the gut microbiota compared to the healthy bowel, with individuals having IBD showing reduced bacterial diversity compared to healthy individuals.[1,2]Indeed, the currently accepted paradigm concerning the pathogenesis of IBD involves an aberrant immunological response to the intestinal microbiota in a genetically susceptible host.[3] Whether the observed dysbiosis represents "cause" or "effect" remains unanswered. Faecal Microbiota Transplant (FMT) is the infusion of a faecal suspension from a healthy donor into the gastrointestinal tract of a patient with disease, and there is interest in the potential of this technique for modifying the gut microbiome as a potential treatment for IBD.[4]

Since the first descriptions of CD and UC at the beginning of the twentieth century, it has been strongly suspected that the gut microbiota may have a defining role in the pathogenesis of IBD.[5] Early culture-based studies underestimated the complexity of the microbiota, and were hampered by various culture biases and the challenge of enumerating fastidious bacteria with complex growth conditions. With the advent of cheap high-throughput genetic sequencing techniques allied with complex bioinformatics capability, there has been a revolution in our understanding of the composition and function of the colonic microbiome. As a result of studies on the microbiome both in patients with IBD and animal models, we know that patients with IBD (either CD or UC) have, at the phylum level, a reduction in Firmicutes and a relative increase in Proteobacteria.[2,6]

Accumulating data suggests that alteration in the gut microbiome plays a central role in driving UC; datasets highlighting the importance of *Roseburia hominis*[7], *Faecalibacterium prausnitzii*[8] and *Akkermansia muciniphila*[9] driving inflammation in UC have been published. Attempts to alter the microbiome with probiotics, whilst disappointing in CD, have shown some promise in UC.[10,11]Since the first exploratory use of FMT to treat UC in 1989[12], numerous case series have been published demonstrating encouraging efficacy signals.[13]This has led to investigators testing FMT as a treatment for UC and to date there have been four randomised controlled trials (RCTs) of FMT for the treatment of UC.[14] The first was a trial of fresh and frozen enemas which were delivered weekly for 6 weeks, water being used as placebo.[15] There was a significant increase in remission at the primary endpoint in the active versus placebo arm. In the second study, fresh FMT by naso-jejunal delivery was used and autologous stool was used as placebo.[16] Although the results favoured active treatment, they were not statistically significant. The last two RCTs were reported in 2017[17,18]and both used frozen

For peer review only - http://bmjopen.bmj.com/site/about/guidelines.xhtml

FMT from pooled donors compared to saline placebo and involved intensive treatment regimens. Both achieved statistically significant results in favour of active treatment, with remission rates comparing active and placebo of 32% versus 9%[18] and 27% versus 8% respectively[17].

As a result of this recent work, there is great interest in FMT as a possible treatment for UC, but the optimal route of delivery remains unknown. In this pilot study, we will compare naso-gastric (NG) versus colonoscopic (COLON) delivery of FMT, using an intensive treatment regimen, in patients with active UC to investigate the optimum route of FMT delivery for the treatment of UC. We will assess the effectiveness and acceptability of the two routes of FMT delivery, and whether FMT by either NG or COLON is suitable to take forward to a full randomised controlled trial against placebo, and whether such a trial is feasible. Here we describe the protocol for the STOP-COLITIS pilot study.

For peer review only

METHODS AND ANALYSIS

Study design

A prospective, multi-centre open-label, randomised pilot study to assess two possible routes of FMT delivery for the treatment of UC. Thirty patients with active UC will be randomised to receive FMT via either the naso-gastric route or by direct delivery to the colon. Patients will be recruited from three hospitals in the United Kingdom (Queen Elizabeth Hospital, Birmingham; St Mark's Hospital, London; and Glasgow Royal Infirmary).

At the end of the pilot, the data will be reviewed by an Independent Oversight Committee (IOC), consisting of two consultant gastroenterologists and a professor of medical statistics and clinical trials, who, according to the pre-specified STOP/GO criteria, (see below) will decide (1) which route of FMT administration (NG or COLON) is most appropriate to investigate in the full randomised double-blind, placebo-controlled trial, and (2) whether it is feasible to proceed to the main randomised controlled trial.

Sample size

As this is a pilot study, no formal sample size calculation was undertaken as the study is not designed or powered to detect a statistically significant difference in efficacy between the two FMT methods of delivery. The recruitment target for this study is 30 patients.

Aims and objectives

The aims of this pilot study are:

i) to determine which FMT administration route (NG or COLON) should be investigated in a randomised double-blind, placebo-controlled trial; and

ii) to determine if a full randomised controlled trial is feasible.

In order to achieve these aims, the pilot study has the following clinical (quantitative), qualitative and mechanistic objectives.

Clinical objectives

To assess:

- Whether FMT by the NG route induces clinical response in patients with active UC;
- Whether FMT by the colonic route induces clinical response in patients with active UC;
- Tolerability and safety;

For peer review only - http://bmjopen.bmj.com/site/about/guidelines.xhtml

- Which route of FMT delivery (if any) is suitable to investigate in the RCT.

Qualitative objectives

To assess:

- Patient acceptability of FMT (NG);
- Patient acceptability of FMT (COLON).

Mechanistic objectives

To assess:

- Whether FMT by either route is associated with a change in faecal calprotectin as a surrogate marker of colonic inflammation;
- Changes in the colonic microbiome and metabolome (short chain fatty acids (SCFA)) induced by FMT via each route;
- Reduction in C-reactive protein (CRP);
- Engraftment of donor microbiota in recipients by culturing donor stool to strain level.

Other objectives

- Effect of diet (donors) on microbiome;
- Effect of time from stool donation to treatment on success of FMT.

Outcome measures

The primary outcome will be a composite assessment of both qualitative and quantitative data relating to efficacy, acceptability, safety and mechanistic outcomes.

Clinical outcome measures

- Clinical response (primary measure of efficacy) defined as ≥3 point reduction in the full Mayo score from randomisation to week 8, and 30% reduction from randomisation and at least 1 point reduction of rectal bleeding sub-score or an absolute rectal bleeding sub-score of 0 or 1;
- Time to clinical response (where clinical response is defined as ≥2 point reduction in partial Mayo);
- Clinical remission at week 8 (full Mayo score of ≤2, with no sub-score >1);
- Participant's weight at week 8 and week 12;
- Quality of Life (QoL) using the generic Short-Form 36 (SF-36) and the disease specific Inflammatory Bowel Disease Questionnaire (IBDQ) at week 8 and week 12;

For peer review only - http://bmjopen.bmj.com/site/about/guidelines.xhtml

- Adherence to FMT;
- Adverse events (AE) and serious adverse events (SAE).

Qualitative data

Patient acceptability will be assessed through qualitative research interviews.

Mechanistic outcome measures

- Faecal calprotectin;
- Measures of microbiome (faecal and mucosal);
- Mucosal healing;
- Urinary metabolome (SCFA);
- CRP;
- Donor faecal culture.

Other outcome measures

- Association between the donor's dietary profile and microbiome;
- Time, i.e. number of days, from donor stool processing to treatment for association between efficacy and freezer life of FMT.

Donor sample acquisition and processing

Donors will be recruited following advertisement from healthy un-related anonymous individuals living in Birmingham. We are excluding healthcare workers due to their potential exposure to microbes affecting the microbiome. Donors must be ≥18 and <50 years of age, have a normal morning bowel habit, have a normal body mass index (BMI; ≥18.5 and ≤25), be non-smokers (not smoked for at least 12 months) and have no recent history of diarrhoea or rectal bleeding. Potential donors will undergo rigorous screening using a health-screening questionnaire. Those who are eligible following this screening will be consented for the donation process and have blood and faecal samples taken to test for transmissible pathogens in accordance with UK, AGA and European guidelines.[19–21]Individuals who pass the screening process will be invited to donate morning faecal samples for 10 days and to deliver these for processing within 6 hours of defaecation. At first donation, they will also be asked to complete the EPIC-Norfolk food frequency questionnaire.[22]

Donations will be processed and stored under an MHRA manufacturing licence at the University of Birmingham Microbiome Treatment Centre. Donations will be prepared in saline with 10% glycerol added as a cytoprotectant. Samples will then be stored frozen at -80°C for up to 24 weeks. Samples

For peer review only - http://bmjopen.bmj.com/site/about/guidelines.xhtml

will be dispatched for use at each site as required. An aliquot from each donated sample will be kept for analysis in the event of adverse events occurring as a result of FMT and samples from each donation period will be sent for genetic sequencing and metabolic analysis. At the end of each 10 day donation period, donors will receive financial compensation and will undergo an exit health questionnaire and be asked to provide a further stool sample to test for pathogens.

Participants

Inclusion criteria

- Adult patients (aged between 16 and 70 years) with clinically confirmed UC for at least 12 weeks prior to the screening visit;
- Partial Mayo score of ≥4 and ≤8 despite stable disease maintenance treatment with 5-aminosalicylates with or without immunomodulators, or on no treatment;
- Rectal bleeding subscore of ≥1 on the partial Mayo;
- Able to give written, signed informed consent.

Exclusion criteria

- Stool positive for *Clostridium difficile* or infection by either PCR or ELISA;
- Positive for Hepatitis A/B/C, and/or Human Immunodeficiency Virus (HIV) infection;
- Antibiotics in the preceding 12 weeks prior to date of the screening visit;
- Systemic/topical steroids in the preceding 2 weeks prior to the date of the screening visit;
- Biologics in the preceding 12 weeks prior to the date of screening visit;
- Commercial probiotics and prebiotics in the preceding 12 weeks prior to the date of the screening visit;
- On oral nutritional supplements or enteral/parenteral nutrition in the preceding 4 weeks prior to the date of the screening visit;
- Pregnant or lactating;
- Not willing to take appropriate contraceptive measures to prevent pregnancy during trial participation.

Participant enrolment

Potentially eligible patients who express an interest in participating in the trial will be consented via a two-stage consent process. Participant information sheets will be provided to facilitate the consent process.

For peer review only - http://bmjopen.bmj.com/site/about/guidelines.xhtml

Registration and screening visit

The first stage will involve consent for trial-specific screening activities, and consent to collect stool and urine samples for the mechanistic sub-studies. Patients will undergo basic physiological assessments (pulse, blood pressure, temperature, height and weight) and baseline blood tests. They will be provided with a diary to record bowel symptoms (so that the partial Mayo score can be calculated at the randomisation visit), stool sample kits and bowel preparation kits (Moviprep®). They will be asked to return the *C. difficile* stool sample as soon as possible, so that the result is available prior to the randomisation visit.

Following the screening visit, the qualitative researcher will arrange for an interview with the patient to take place prior to their randomisation visit (see below). The research team at the hospital will contact the patient to notify them of their stool result. If they tested negative for *C.difficile*, they will be invited to attend the randomisation visit. Instructions will be given on when to take the bowel preparation, and they will be asked to collect a stool sample on the same day prior to taking the bowel preparation, which they will bring to the randomisation visit.

Randomisation visit

The second stage is consent for entry into the trial. Following confirmation of all eligibility criteria and consent to randomisation, basic physiological assessments will be undertaken, blood test results will be checked, a urine sample will be taken for pregnancy testing in women and metabolomics and the partial Mayo score will be calculated from diaries. The patient will be asked to complete the baseline QoL questionnaires; IBDQ and SF-36. All patients will have a colonoscopy to assess disease, so that a full Mayo score can be calculated, and to collect mucosal biopsies.

Randomisation

Patients will be randomised at the level of the individual in a 1:1 ratio to either NG or COLON delivery of FMT. Randomisation will be provided by a computer-generated program at the Birmingham Clinical Trials Unit (BCTU). A minimisation algorithm will be used to ensure balance in the treatment allocation over the following variables:

- Partial Mayo score (4-5 or 6-8);
- Current smoking status (current smoker: yes or no (not smoked for the past 12 months));

For peer review only - http://bmjopen.bmj.com/site/about/guidelines.xhtml

A 'random element' will be included in the minimisation algorithm, so that each patient has a probability (unspecified here), of being randomised to the opposite treatment that they would have otherwise received.

Interventions

Before the initial FMT treatment, all patients will receive standard bowel preparation: 2 litres of reconstituted Moviprep® solution within the 24 hours prior to procedure.

Naso-gastric treatment (NG)

Patients randomised to NG treatment will be pre-treated with a proton pump inhibitor (lansoprazole 30mg) and a prokinetic agent (domperidone 10mg) at least 30 minutes prior to each FMT infusion to reduce gastric acid secretion and prevent the risk of gastro-oesophageal regurgitation. Naso-gastric tubes (NGT) will be positioned and checked for correct position as per local protocol. Following thawing at room temperature, 50ml thawed FMT containing 30g faeces will be infused. Patients will receive a single dose of 2mg loperamide after each infusion to promote retention of the infusion. Following the first treatment, patients will return for the next three days for further treatment following an overnight fast. It will be the individual's choice whether they wish to retain the NGT for the 4 day treatment course or have the tube removed and re-passed every day. On the last day of treatment, the NGT will be removed to be replaced for the second course of treatment. At week 4, patients will return for a further 4 FMT infusions over 4 consecutive days following a fast from midnight.

In summary, patients will receive 30g FMT in 50ml aliquots for NG administration each day for 4 days at the start of the trial (starting on the day of randomisation), and then again for 4 days in week 4 (total FMT dose 240g).

Colonoscopic treatment (COLON)

Patients randomised to COLON treatment will receive a thawed 250ml aliquot of FMT (containing 150g faeces) on the day of randomisation. This will be delivered to the colon using a spray catheter, with 125ml sprayed into the caecum to treat the right side of the bowel and the remaining 125ml sprayed directly onto the rest of the colon. Following treatment, patients will receive a single dose of loperamide. Patients will return on a weekly basis to receive 100ml enema up to week 7 containing 50ml of saline and 30g faeces, followed by loperamide each time.

For peer review only - http://bmjopen.bmj.com/site/about/guidelines.xhtml

In summary, patients will receive 150g of stool in 250ml for colonic administration on the day of randomisation followed by 30g of stool in 100ml in normal saline for administration by enema for 7 weeks (total FMT dose 360g).

Data Collection

All patients will be followed weekly up to week 8, and then again at week 12. At each visit, the partial Mayo score will be calculated from the patient diaries, and medication use and any adverse events occurring will be recorded. Stool samples will be collected at weeks 2, 4, 6, 8 and 12, and blood samples will be taken for CRP assessment at weeks 4, 6 and 8. At weeks 8 and 12, physiological data, a urine sample and the SF-36 and IBDQ QoL questionnaires will also be collected. At week 8, a flexible sigmoidoscopy will be undertaken to calculate the full Mayo Score. See figure 1.

Figure 1 Trial schema and data collection outline

For peer review only

Qualitative Research: Patient and clinician experience and acceptability of FMT and trial processes

A comparison of the acceptability of the two routes of FMT being used in this study is key to the outcome of this pilot study. A qualitative researcher will conduct interviews with patients participating in both arms of the trial. Each patient will take part in a semi-structured interview at two time points; following the screening visit prior to randomisation and after the 12-week follow up visit. These one-to-one interviews will take place either in person, by telephone or Skype, as preferred by the patients. The initial interview will focus on participants' experience of UC and treatment to date, along with their understanding and expectations for FMT and the pilot study. A background questionnaire collecting socio-demographic and clinical details will also be administered. During the follow up interview, patients' actual experience within the pilot study, including that of FMT (by NG and COLON) and of related trial processes and procedures will be explored. Interviews will also gather data on patient perspectives regarding important impacts and outcomes of FMT, thus providing the opportunity to compare these with the outcome measures selected for inclusion in the pilot and (later) full RCT. Further interviews with patients who withdraw from FMT treatment early and with a small sample of patients who decline consent to participate following screening (should this happen), will provide in-depth understanding of these decisions. Interviews will also be conducted with staff at the pilot sites to review their experience of trial conduct e.g. the acceptability and logistics of complex trial procedures within clinical environments.

Data from this qualitative research will contribute to an assessment of patient and clinician experience and acceptability of FMT within the pilot context, and along with recruitment and drop-out rates, and data regarding clinical response, this will inform a decision about whether the NG or COLON route of delivery is taken forward to the full RCT. The qualitative data will also contribute to the refinement and optimisation of trial processes prior to the full RCT.

Analysis of qualitative data

Interviews will be recorded with the consent of participants and transcribed clean verbatim for analysis. Analysis will be conducted with reference to recordings, transcripts and field notes taken at the time of data collection. A thematic analysis of content will be informed by the Framework analytical approach.[23] Following initial familiarisation with the interview data, development of thematic frameworks and data coding will proceed in an iterative manner. Data collection and analysis will run concurrently so that emergent analytical themes can inform further data collection, and in particular comparative analytical questioning between patients allocated to NG or COLON.

For peer review only - http://bmjopen.bmj.com/site/about/guidelines.xhtml

Mechanistic studies

There will be two aspects to mechanistic studies undertaken as part of this trial; non-invasive assessment of UC activity and assessment of patient's microbiome response to FMT in comparison with that of donor microbiome at baseline. The study will also investigate donor diet with regard to baseline microbiome and treatment effect.

Non-invasive assessment of UC activity

This will be undertaken using the serum biomarker CRP and faecal calprotectin which will be measured using a standard protocol at one central laboratory in Glasgow.

Microbiome related assays

16S ribosomal DNA sequencing and metagenomics

16S ribosomal DNA sequencing will be performed on all samples collected during the study: donor faeces (fresh and frozen from day 1 and day 10 donations), faeces from the recipient at baseline, weeks 2, 4, 6, 8, and 12 and mucosal biopsies taken from the right colon, left colon and rectum at baseline and from left colon and rectum at week 8. Together these samples will be compared to clinical data, and correlations found with faecal calprotectin, in order to find the samples most likely to be biologically informative as a marker of disease remission. Additionally, and based on the results of the initial 16S analysis, a shotgun metagenomics approach will be undertaken on around 200 selected samples to give insights regarding functional enrichment. For a certain subset of samples where additional strain resolution is required (e.g. recovery of complete genomes of interest) or linkage between extra-chromosomal elements such as plasmids and phages, further samples will be subject to long-read sequencing.

Major bacterial metabolites and metabolomics on faecal and urine samples

Major bacterial metabolites including faecal short and medium chain fatty acids previously implicated in the aetiology and mucosal inflammation of IBD will be measured using gas chromatography and other assays as described previously.[24–26] Analysis of short chain fatty acids in the samples will be carried out on faecal samples from the donors on day 1 and day 10 and on patients at baseline, week 8 and week 12.

Donor assessment of dietary intake

The habitual dietary pattern of the donors will be assessed using the validated food frequency questionnaire as used in the EPIC-Norfolk study in the UK.[27]Data from the EPIC questionnaires will be

For peer review only - http://bmjopen.bmj.com/site/about/guidelines.xhtml

transferred to Glasgow for analysis. Energy, macronutrient and fibre intake will be estimated and expressed in nutrient ranks and quartiles and will also be compared against Department of Health recommendations and UK National Diet and Nutrition Survey results.

Analysis of outcome measures

The primary outcome from the pilot study will be a recommendation about which route of administration (NG or COLON) to take forward to the main RCT, and whether a full RCT is feasible. The decision will be based on assessing the two methods of delivery using a composite assessment of both qualitative and quantitative data on efficacy using clinical response, acceptability and safety.

The primary comparison will comprise of those randomised to FMT by the NG route versus those randomised to FMT by the COLON route. All analyses will be based on the intention to treat principle. Data will be presented as summary statistics and differences between groups will be presented with 95% confidence intervals (CI) from 2-sided tests. No formal hypothesis testing will be undertaken and no p-values will be presented.

Primary outcome measure of efficacy for STOP/GO

The primary measure of efficacy for the STOP/GO is clinical response at week 8. The number and percentage of patients achieving a clinical response in the two treatment groups will be reported along with the 95% CI. A log-binomial model will be fitted to obtain a relative risk and 95% CI, adjusting for the minimisation variable smoking status and baseline full Mayo score.

Secondary Clinical Outcomes Measures for STOP/GO

The number and percentage of patients achieving clinical remission in the two treatment groups will be reported. A log-binomial model will be fitted to obtain a relative risk and 95% CI, adjusting for the minimisation variables: smoking status and baseline partial Mayo score. Time to clinical response (based on the partial Mayo score) will be presented graphically as a Kaplan-Meier plot. A Cox proportional hazards regression model will be fitted to obtain a hazard ratio and 95% CI. Continuous data (e.g. weight, QoL scores) will be summarised using means and standard deviations presented at each time point (baseline, week 8 and week 12). The week 8 and week 12 data will be compared by treatment group using linear regression models with the minimisation variables and baseline values included in the model, with the mean differences and 95% CI presented.

For peer review only - http://bmjopen.bmj.com/site/about/guidelines.xhtml

Tolerability will be assessed quantitatively using adherence. A patient will be considered adherent if they receive at least 70% of their intended FMT dose. The number and percentage of patients who are deemed adherent to the FMT treatment in the two treatment groups will be reported along with the 95% CIs.

Adverse event and SAE data will be tabulated. For each treatment group, the number of adverse events and the number and percentage of patients experiencing an adverse event will be reported. Data on SAEs will be reported in the same way.

STOP/GO guidelines

At the end of the pilot, the IOC will be convened to review the data. This group will make a recommendation to the Trial Management Group on (1) which route (if any) of FMT is appropriate to take forward to the full trial and (2) whether it is feasible to proceed to the full RCT.

To determine whether we will proceed forward to the main RCT, a pragmatic review of the pilot data in terms of assessing treatment efficacy (based on clinical response), tolerability, and patient acceptability will be undertaken. STOP/GO guidelines will be used to determine whether to proceed forward to the main RCT, and we propose then to take forward the preferred method of FMT delivery into a randomised double-blind placebo-controlled trial with a clinical efficacy outcome (clinical remission).

The first STOP/GO decision will be on which route of FMT to take forward to the full RCT. The pilot is not powered to show differences in the two modes of delivery (NG or COLON); therefore a decision or recommendation on which route to use in the main trial requires expert judgement and cannot be made based on a purely numerical process. The following data will be reviewed by the IOC:

- The proportion of patients who achieve a clinical response following FMT by the NG route†;
- The proportion of patients who achieve a clinical response following FMT by the COLON†;
- Whether FMT by either route is associated with a change in faecal calprotectin;
- Whether FMT by either route is associated with changes in the colonic microbiome and metabolome;
- Tolerability and safety for each route;
- Patient acceptability of FMT by the NG route through the qualitative interviews (including advantages and disadvantages of the NG route);
- Patient acceptability of FMT by the COLON route through the qualitative interviews (including

For peer review only - http://bmjopen.bmj.com/site/about/guidelines.xhtml

advantages and disadvantages of the COLON route).

† A threshold of achieving a clinical response in around 40% of patients treated may be used. This threshold will be agreed after discussions by the Trial Management Group and the IOC prior to any data analysis being performed.

Following review of the above data, whether the IOC can recommend a route to take forward to the main trial, will form the basis of the first STOP/GO decision.

a) IOC unable to recommend one particular route as neither route felt to be satisfactory. DECISION: Do not proceed to main RCT.

b) IOC recommends a route of FMT delivery for use in the main RCT. DECISION: Proceed to STOP/GO decision stage 2 of pilot.

Once the route of FMT delivery has been selected, a second STOP/GO assessing feasibility will be used to determine whether to proceed to the main RCT. This will be based on the following:

1) That the IOC are able to recommend a route.

2) That the recruitment of the 30 patients in the pilot averages 0.7 patients per week in each open site.

3) That 10 of the 15 patients in the route cohort selected for the main study received at least 70% of their intended FMT dose. For example, 70% of the NG route (240g) is 168g, which requires the patient to receive at least 6 infusions (30g; 6 out of the 8 FMT infusions i.e. patient can miss no more than two doses); and 70% of the COLON route (360g) is 252g, which requires the patient to receive the first infusion by colonoscopy (150g) and then at least 4 out of 7 of the enema infusions (30g) i.e. patient can miss no more than three doses.

4) That the IOC have not identified any safety concerns.

Following review of the above data, whether the IOC are able to recommend that a full RCT is feasible will form the basis of the second STOP/GO decision.

a) IOC consider a RCT unfeasible. DECISION: Do not proceed to main RCT.

b) IOC consider a RCT feasible. DECISION: Provide report to funder with recommendation of IOC, in order for a final decision to be made on progression to the full RCT.

For peer review only - http://bmjopen.bmj.com/site/about/guidelines.xhtml

Patient and Public Involvement

Our protocol was developed in consultation with a research PPI group referred to as the Clinical Research Ambassador Group (CRAG), based at University Hospital NHS Foundation Trust. One of the co-applicants is a patient with IBD and also Chairperson for the West Midlands Group of Crohn's and Colitis UK and she helped develop the proposal. The PPI has strong links with CCUK and this will significantly aid dissemination of findings nationally to patients, relatives and health professionals.

As we are aware that acceptability of FMT in UC has not been extensively studied we designed the pilot study to investigate patient acceptability of the therapy by regular questionnaires, individual interviews and group discussions. We conducted a survey of our own IBD patients from the outpatient department at University Hospital Birmingham. Of the 74 patient surveyed the vast majority of patients would accept FMT as a treatment for IBD and over 80% would consider involvement in a trial of FMT in IBD. Qualitative interviews with patients will be been conducted at baseline and at the end of the study and these findings are an outcome measure of the study. Patients recruited to the study will not necessarily directly assist with the recruitment of other patients.

Ethics And Dissemination

The trial will be performed in accordance with the recommendations guiding physicians in biomedical research involving human subjects, adopted by the 18th World Medical Association General Assembly, Helsinki, Finland, June 1964, amended at the 48th World Medical Association General Assembly, Somerset West, Republic of South Africa, October 1996.

The trial will be conducted in accordance with the Research Governance Framework for Health and Social Care, the applicable UK Statutory Instruments, (which include the Medicines for Human Use Clinical Trials 2004 and subsequent amendments and the General Data Protection Regulation (GDPR) (EU) 2016/679 and Human Tissue Act 2008) and Guidelines for Good Clinical Practice (GCP). This trial will be carried out under a Clinical Trial Authorisation in accordance with the Medicines for Human Use Clinical Trials regulations.

Ethical approved for this study has been obtained from the East Midlands-Nottingham Research Ethics Committee (REC 17/EM/0274).

Publication policy

At the end of the study, a meeting will be held to allow discussion of the main results among the collaborators prior to publication. The success of the study will depend entirely on the wholehearted

For peer review only - http://bmjopen.bmj.com/site/about/guidelines.xhtml

collaboration of a large number of doctors, nurses and others. For this reason, the chief credit for the main results will be given not only to the central supervisory committees and/or organisers, but to all those who have collaborated in the trial.

The findings will be reported at national and international Gastroenterology meetings and published in peer-reviewed journals.

Authors' Contributions

MNQ, MY, JS, NS, PH, VLM, AH, DG, RH, NI, LM, SL, CH, NC, and TI were all involved in developing the clinical protocol and authoring the manuscript , with MNQ being the lead author, TI is the Chief Investigator. NI is the senior statistician on the project. She was involved in the trial design, protocol development and led the development of the proposed statistical analyses. CAH is the trial statistician and was involved in protocol development and produced the statistical analysis plan for this study. SL is the trial co-ordinator and with LM developed the trial management and governance aspects of the manuscript. KG, NL, CQ contributed the mechanistics and bio-informatics expertise. JM and CM authored the qualitative research section of the manuscript. We thank our patient advisor NC for her help with developing this protocol. We used the SPIRIT checklist when writing this protocol.[28]

Funding Statement

This study is funded by the National Institute for Health Research (NIHR) as part of the Efficacy and Mechanism Evaluation (EME) programme.

Competing Interests Statement

None

For peer review only - http://bmjopen.bmj.com/site/about/guidelines.xhtml

REFERENCES

1. Qin, J. *et al.* A human gut microbial gene catalogue established by metagenomic sequencing. *Nature* **464**, 59 (2010).
2. Gerasimidis, K. *et al.* Decline in presumptively protective gut bacterial species and metabolites are paradoxically associated with disease improvement in pediatric Crohn's disease during enteral nutrition. *Inflamm. Bowel Dis.* **20**, 861–871 (2014).
3. de Souza, H. S. P. & Fiocchi, C. Immunopathogenesis of IBD: current state of the art. *Nat. Rev. Gastroenterol. Hepatol.* **13**, 13–27 (2016).
4. Anderson, J. L., Edney, R. J. & Whelan, K. Systematic review: faecal microbiota transplantation in the management of inflammatory bowel disease. *Aliment. Pharmacol. Ther.* **36**, 503–516 (2012).
5. Sartor, R. B. Microbial influences in inflammatory bowel diseases. *Gastroenterology* **134**, 577–594 (2008).
6. Kostic, A. D., Xavier, R. J. & Gevers, D. The microbiome in inflammatory bowel disease: current status and the future ahead. *Gastroenterology* **146**, 1489–1499 (2014).
7. Machiels, K. *et al.* A decrease of the butyrate-producing species Roseburia hominis and Faecalibacterium prausnitzii defines dysbiosis in patients with ulcerative colitis. *Gut* **63**, 1275–1283 (2014).
8. Varela, E. *et al.* Colonisation by Faecalibacterium prausnitzii and maintenance of clinical remission in patients with ulcerative colitis. *Aliment. Pharmacol. Ther.* **38**, 151–161 (2013).
9. Manichanh, C., Borruel, N., Casellas, F. & Guarner, F. The gut microbiota in IBD. *Nat. Rev. Gastroenterol. Hepatol.* **9**, 599–608 (2012).
10. Sood, A. *et al.* The probiotic preparation, VSL#3 induces remission in patients with mild-to-moderately active ulcerative colitis. *Clin. Gastroenterol. Hepatol. Off. Clin. Pract. J. Am. Gastroenterol. Assoc.* **7**, 1202–1209, 1209.e1 (2009).

For peer review only

For peer review only - http://bmjopen.bmj.com/site/about/guidelines.xhtml

11. Tursi, A. *et al.* Treatment of Relapsing Mild-to-Moderate Ulcerative Colitis With the Probiotic VSL#3 as Adjunctive to a Standard Pharmaceutical Treatment: A Double-Blind, Randomized, Placebo-Controlled Study. *Am. J. Gastroenterol.* **105**, 2218 (2010).

12. Bennet, J. D. & Brinkman, M. Treatment of ulcerative colitis by implantation of normal colonic flora. *Lancet Lond. Engl.* **1**, 164 (1989).

13. Costello, S. P. *et al.* Systematic review with meta-analysis: faecal microbiota transplantation for the induction of remission for active ulcerative colitis. *Aliment. Pharmacol. Ther.* **46**, 213–224 (2017).

14. Paramsothy, S. *et al.* Faecal Microbiota Transplantation for Inflammatory Bowel Disease: A Systematic Review and Meta-analysis. *J. Crohns Colitis* **11**, 1180–1199 (2017).

15. Moayyedi, P. *et al.* Fecal Microbiota Transplantation Induces Remission in Patients With Active Ulcerative Colitis in a Randomized Controlled Trial. *Gastroenterology* **149**, 102-109.e6 (2015).

16. Rossen, N. G. *et al.* Findings From a Randomized Controlled Trial of Fecal Transplantation for Patients With Ulcerative Colitis. *Gastroenterology* **149**, 110-118.e4 (2015).

17. Paramsothy, S. *et al.* Multidonor intensive faecal microbiota transplantation for active ulcerative colitis: a randomised placebo-controlled trial. *Lancet Lond. Engl.* **389**, 1218–1228 (2017).

18. Costello, S. P. *et al.* Effect of Fecal Microbiota Transplantation on 8-Week Remission in Patients With Ulcerative Colitis: A Randomized Clinical Trial. *JAMA* **321**, 156–164 (2019).

19. Letter from the Presidents of the Infectious Disease Society of America, the American Gastroenterological Association, the American Society for Gastrointestinal Endoscopy et al., Current Consensus Guidance on Donor Screening and Stool Testing for FMT, July 5, 2013, http://www.gastro.org/research/Joint_Society_FMT_Guidance.pdf (accessed December, 2017).

20. Cammarota, G. *et al.* European consensus conference on faecal microbiota transplantation in clinical practice. *Gut* gutjnl-2016-313017 (2017). doi:10.1136/gutjnl-2016-313017

21. Mullish, B. H. *et al.* The use of faecal microbiota transplant as treatment for recurrent or refractory Clostridium difficile infection and other potential indications: joint British Society of

For peer review only

For peer review only - http://bmjopen.bmj.com/site/about/guidelines.xhtml

Gastroenterology (BSG) and Healthcare Infection Society (HIS) guidelines. *Gut* **67**, 1920–1941 (2018).

22. Moskal, A. *et al.* Nutrient Patterns and Their Food Sources in an International Study Setting: Report from the EPIC Study. *PLoS ONE* **9**, (2014).
23. *Ritchie, J., Spencer, L. & O'Connor, W. Carrying out qualitative analysis in: Qualitative Research Practice. J Ritchie and L Lewis (eds) London: Sage (2003).*
24. Kamleh, A. *et al.* Metabolomic profiling using Orbitrap Fourier transform mass spectrometry with hydrophilic interaction chromatography: a method with wide applicability to analysis of biomolecules. *Rapid Commun. Mass Spectrom. RCM* **22**, 1912–1918 (2008).
25. Creek, D. J., Jankevics, A., Burgess, K. E. V., Breitling, R. & Barrett, M. P. IDEOM: an Excel interface for analysis of LC-MS-based metabolomics data. *Bioinforma. Oxf. Engl.* **28**, 1048–1049 (2012).
26. Creek, D. J. *et al.* Toward global metabolomics analysis with hydrophilic interaction liquid chromatography-mass spectrometry: improved metabolite identification by retention time prediction. *Anal. Chem.* **83**, 8703–8710 (2011).
27. Welch, A. A., Luben, R., Khaw, K. T. & Bingham, S. A. The CAFE computer program for nutritional analysis of the EPIC-Norfolk food frequency questionnaire and identification of extreme nutrient values. *J. Hum. Nutr. Diet. Off. J. Br. Diet. Assoc.* **18**, 99–116 (2005).
28. Chan, A.-W. *et al.* SPIRIT 2013 Statement: Defining Standard Protocol Items for Clinical Trials. *Ann. Intern. Med.* **158**, 200–207 (2013).

For peer review only

For peer review only - http://bmjopen.bmj.com/site/about/guidelines.xhtml

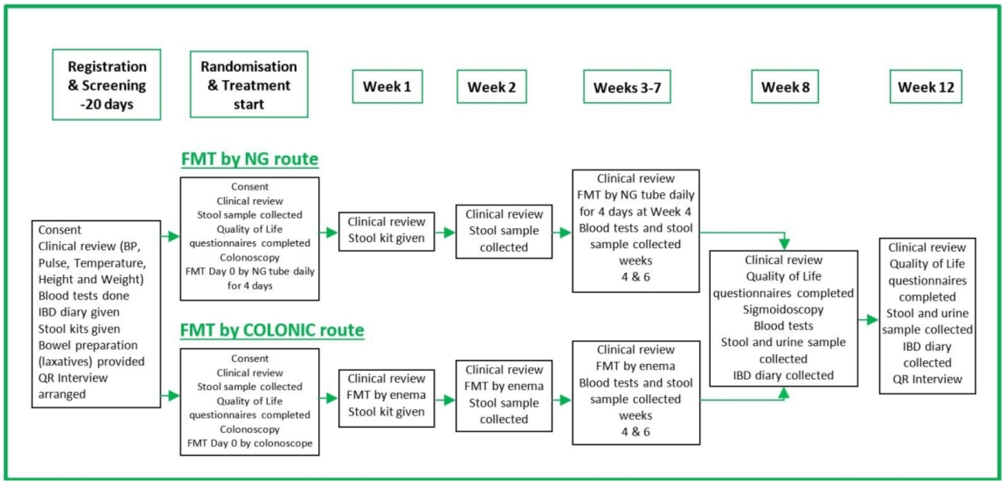

Figure 1 Trial schema and data collection outline

422x201mm (96 x 96 DPI)

Reporting checklist for protocol of a clinical trial.

Based on the SPIRIT guidelines.

		Reporting Item	Page Number
Title	#1	Descriptive title identifying the study design, population, interventions, and, if applicable, trial acronym	1
Trial registration	#2a	Trial identifier and registry name. If not yet registered, name of intended registry	3
Trial registration: data set	#2b	All items from the World Health Organization Trial Registration Data Set	3
Protocol version	#3	Date and version identifier	3
Funding	#4	Sources and types of financial, material, and other support	3
Roles and responsibilities: contributorship	#5a	Names, affiliations, and roles of protocol contributors	18
Roles and responsibilities: sponsor contact information	#5b	Name and contact information for the trial sponsor	19
Roles and responsibilities:	#5c	Role of study sponsor and funders, if any, in study design; collection, management, analysis, and interpretation of	17

For peer review only

sponsor and funder		data; writing of the report; and the decision to submit the report for publication, including whether they will have ultimate authority over any of these activities	
Roles and responsibilities: committees	#5d	Composition, roles, and responsibilities of the coordinating centre, steering committee, endpoint adjudication committee, data management team, and other individuals or groups overseeing the trial, if applicable (see Item 21a for data monitoring committee)	18
Background and rationale	#6a	Description of research question and justification for undertaking the trial, including summary of relevant studies (published and unpublished) examining benefits and harms for each intervention	4
Background and rationale: choice of comparators	#6b	Explanation for choice of comparators	4
Objectives	#7	Specific objectives or hypotheses	6
Trial design	#8	Description of trial design including type of trial (eg, parallel group, crossover, factorial, single group), allocation ratio, and framework (eg, superiority, equivalence, non-inferiority, exploratory)	6
Study setting	#9	Description of study settings (eg, community clinic, academic hospital) and list of countries where data will be collected. Reference to where list of study sites can be obtained	6

For peer review only

Eligibility criteria	#10	Inclusion and exclusion criteria for participants. If applicable, eligibility criteria for study centres and individuals who will perform the interventions (eg, surgeons, psychotherapists)	9
Interventions: description	#11a	Interventions for each group with sufficient detail to allow replication, including how and when they will be administered	11
Interventions: modifications	#11b	Criteria for discontinuing or modifying allocated interventions for a given trial participant (eg, drug dose change in response to harms, participant request, or improving / worsening disease)	11
Interventions: adherance	#11c	Strategies to improve adherence to intervention protocols, and any procedures for monitoring adherence (eg, drug tablet return; laboratory tests)	n/a
Interventions: concomitant care	#11d	Relevant concomitant care and interventions that are permitted or prohibited during the trial	9
Outcomes	#12	Primary, secondary, and other outcomes, including the specific measurement variable (eg, systolic blood pressure), analysis metric (eg, change from baseline, final value, time to event), method of aggregation (eg, median, proportion), and time point for each outcome. Explanation of the clinical relevance of chosen efficacy and harm outcomes is strongly recommended	7
Participant timeline	#13	Time schedule of enrolment, interventions (including any	12

For peer review only

		run-ins and washouts), assessments, and visits for participants. A schematic diagram is highly recommended (see Figure)	
Sample size	#14	Estimated number of participants needed to achieve study objectives and how it was determined, including clinical and statistical assumptions supporting any sample size calculations	6
Recruitment	#15	Strategies for achieving adequate participant enrolment to reach target sample size	n/a
Allocation: sequence generation	#16a	Method of generating the allocation sequence (eg, computer-generated random numbers), and list of any factors for stratification. To reduce predictability of a random sequence, details of any planned restriction (eg, blocking) should be provided in a separate document that is unavailable to those who enrol participants or assign interventions	10
Allocation concealment mechanism	#16b	Mechanism of implementing the allocation sequence (eg, central telephone; sequentially numbered, opaque, sealed envelopes), describing any steps to conceal the sequence until interventions are assigned	10
Allocation: implementation	#16c	Who will generate the allocation sequence, who will enrol participants, and who will assign participants to interventions	10
Blinding (masking)	#17a	Who will be blinded after assignment to interventions (eg,	n/a

For peer review only

		trial participants, care providers, outcome assessors, data analysts), and how	
Blinding (masking): emergency unblinding	#17b	If blinded, circumstances under which unblinding is permissible, and procedure for revealing a participant's allocated intervention during the trial	n/a
Data collection plan	#18a	Plans for assessment and collection of outcome, baseline, and other trial data, including any related processes to promote data quality (eg, duplicate measurements, training of assessors) and a description of study instruments (eg, questionnaires, laboratory tests) along with their reliability and validity, if known. Reference to where data collection forms can be found, if not in the protocol	15
Data collection plan: retention	#18b	Plans to promote participant retention and complete follow-up, including list of any outcome data to be collected for participants who discontinue or deviate from intervention protocols	15
Data management	#19	Plans for data entry, coding, security, and storage, including any related processes to promote data quality (eg, double data entry; range checks for data values). Reference to where details of data management procedures can be found, if not in the protocol	18
Statistics: outcomes	#20a	Statistical methods for analysing primary and secondary outcomes. Reference to where other details of the statistical analysis plan can be found, if not in the protocol	15

For peer review only

Statistics: additional analyses	#20b	Methods for any additional analyses (eg, subgroup and adjusted analyses)	n/a
Statistics: analysis population and missing data	#20c	Definition of analysis population relating to protocol non-adherence (eg, as randomised analysis), and any statistical methods to handle missing data (eg, multiple imputation)	n/a
Data monitoring: formal committee	#21a	Composition of data monitoring committee (DMC); summary of its role and reporting structure; statement of whether it is independent from the sponsor and competing interests; and reference to where further details about its charter can be found, if not in the protocol. Alternatively, an explanation of why a DMC is not needed	16
Data monitoring: interim analysis	#21b	Description of any interim analyses and stopping guidelines, including who will have access to these interim results and make the final decision to terminate the trial	16
Harms	#22	Plans for collecting, assessing, reporting, and managing solicited and spontaneously reported adverse events and other unintended effects of trial interventions or trial conduct	15
Auditing	#23	Frequency and procedures for auditing trial conduct, if any, and whether the process will be independent from investigators and the sponsor	15
Research ethics approval	#24	Plans for seeking research ethics committee / institutional review board (REC / IRB) approval	17
Protocol	#25	Plans for communicating important protocol modifications	17

For peer review only

amendments		(eg, changes to eligibility criteria, outcomes, analyses) to relevant parties (eg, investigators, REC / IRBs, trial participants, trial registries, journals, regulators)	
Consent or assent	#26a	Who will obtain informed consent or assent from potential trial participants or authorised surrogates, and how (see Item 32)	9
Consent or assent: ancillary studies	#26b	Additional consent provisions for collection and use of participant data and biological specimens in ancillary studies, if applicable	n/a
Confidentiality	#27	How personal information about potential and enrolled participants will be collected, shared, and maintained in order to protect confidentiality before, during, and after the trial	18
Declaration of interests	#28	Financial and other competing interests for principal investigators for the overall trial and each study site	19
Data access	#29	Statement of who will have access to the final trial dataset, and disclosure of contractual agreements that limit such access for investigators	18
Ancillary and post trial care	#30	Provisions, if any, for ancillary and post-trial care, and for compensation to those who suffer harm from trial participation	n/a
Dissemination policy: trial results	#31a	Plans for investigators and sponsor to communicate trial results to participants, healthcare professionals, the public,	18

For peer review only

		and other relevant groups (eg, via publication, reporting in results databases, or other data sharing arrangements), including any publication restrictions	
Dissemination policy: authorship	#31b	Authorship eligibility guidelines and any intended use of professional writers	18
Dissemination policy: reproducible research	#31c	Plans, if any, for granting public access to the full protocol, participant-level dataset, and statistical code	18
Informed consent materials	#32	Model consent form and other related documentation given to participants and authorised surrogates	n/a
Biological specimens	#33	Plans for collection, laboratory evaluation, and storage of biological specimens for genetic or molecular analysis in the current trial and for future use in ancillary studies, if applicable	14

The SPIRIT checklist is distributed under the terms of the Creative Commons Attribution License CC-BY-ND 3.0. This checklist was completed on 25. March 2019 using https://www.goodreports.org/, a tool made by the EQUATOR Network in collaboration with Penelope.ai

For peer review only

BMJ Open

Stop-Colitis pilot trial protocol. A prospective, open-label, randomised pilot study to assess two possible routes of Faecal Microbiota Transplant delivery in patients with ulcerative colitis

Journal:	*BMJ Open*
Manuscript ID	bmjopen-2019-030659.R1
Article Type:	Protocol
Date Submitted by the Author:	04-Jul-2019
Complete List of Authors:	Quraishi, Mohammed Nabil; University of Birmingham, University of Birmingham Microbiome Treatment Centre; University of Birmingham<br>Yalchin, Mehmet; St Marks Hospital<br>Blackwell, Clare; Glasgow Royal Infirmary<br>Segal, Jonathan; St Marks Hospital<br>Sharma, Naveen; Heart of England NHS Foundation Trust, Department of Gastroenterology<br>Hawkey, Peter; University of Birmingham, Institute of Microbiology and Infection<br>McCune, Victoria; University of Birmingham, Institute of Microbiology and Infection; Public Health England Midlands and East Region, Public Health Laboratory Birmingham<br>Hart, Ailsa L.; St Marks Hospital<br>Gaya, Daniel; Glasgow Royal Infirmary<br>Ives, Natalie; University of Birmingham, Clinical Trials Unit<br>Magill, Laura ; University of Birmingham, Clinical Trials Unit<br>Loi, Shrushma; University of Birmingham, Clinical Trials Unit<br>Hewitt, Catherine; University of Birmingham, Clinical Trials Unit<br>Gerasimidis, Konstantinos; University of Glasgow, Human Nutrition<br>Loman, Nicholas; University of Birmingham, Institute of Microbiology and Infection<br>Hansen, Richard; Royal Hospital for Children Glasgow, Department of Paediatric Gastroenterology<br>McMullan, Christel; University of Birmingham, Institute of Applied Health Research<br>Mathers, Jonathan; University of Birmingham, Public Health<br>Quince, Christopher; University of Warwick Warwick Medical School<br>Crees, Nicola; Crohn's and Colitis UK<br>Iqbal, Tariq; University of Birmingham, University of Birmingham Microbiome Treatment Centre; Queen Elizabeth Hospital
**Primary Subject Heading**:	Gastroenterology and hepatology
Secondary Subject Heading:	Medical management
Keywords:	Inflammatory bowel disease < GASTROENTEROLOGY, MICROBIOLOGY, Clinical trials < THERAPEUTICS

SCHOLARONE™
Manuscripts

Title: Stop-Colitis pilot trial protocol. A prospective, open-label, randomised pilot study to assess two possible routes of Faecal Microbiota Transplant delivery in patients with ulcerative colitis

Authors:

Mohammed Nabil Quraishi[1,2], Mehmet Yalchin[3], Clare Blackwell[4], Jonathan Segal[3], Naveen Sharma[5], Peter Hawkey[6], Victoria L McCune[6,7], Ailsa Hart[3], Daniel R Gaya[4], Natalie Ives[8], Laura Magill[8], Shrushma Loi[8], Catherine A Hewitt[8], Konstantinos Gerasimidis[9], Nicholas Loman[6], Richard Hansen[10], Christel McMullan[11], Jonathan Mathers[11], Chris Quince[12], Nicola Crees[13], Tariq Iqbal[1,2]

Affiliations:

[1]University of Birmingham Microbiome Treatment Centre, University of Birmingham, Birmingham

[2]Department of Gastroenterology, University Hospital Birmingham, Birmingham

[3]St Marks Hospital, London

[4]Gastroenterology Unit, Glasgow Royal Infirmary, Glasgow

[5]Department of Gastroenterology, Heart of England Foundation Trust, Birmingham

[6]Institute of Microbiology and Infection, University of Birmingham, Birmingham

[7]Public Health England, Public Health Laboratory Birmingham, Birmingham, United Kingdom

[8]Birmingham Clinical Trials Unit, Institute of Applied Health Research, University of Birmingham, Birmingham

[9]Human Nutrition, School of Medicine, Dentistry and Nursing, Glasgow Royal Infirmary, University of Glasgow, Glasgow

[10]Department of Paediatric Gastroenterology, Royal Hospital for Children, Glasgow

[11]Institute of Applied Health Research, University of Birmingham, Birmingham

[12]Warwick Medical School, Microbiology and Infection, University of Warwick, Warwick

[13]Crohns and Colitis UK, United Kingdom

Corresponding author: Professor Tariq Iqbal; t.h.iqbal@bham.ac.uk

For peer review only

For peer review only - http://bmjopen.bmj.com/site/about/guidelines.xhtml

ABSTRACT

Introduction

Imbalance of the gut microbiome is key to the pathogenesis of ulcerative colitis (UC). Faecal microbiota transplant (FMT) is the transfer of homogenised and filtered faeces from a healthy individual to the gastrointestinal tract of a patient with disease. Published datasets show a positive signal for the use of FMT to treat UC, but the optimal route and dose of FMT remains unanswered.

Methods and analysis

This prospective, multi-centre open-label, randomised pilot study will assess two possible routes of FMT delivery, via the naso-gastric route or by delivery to the colon, in 30 patients with active UC recruited from three sites in the UK. Stool will be collected from healthy screened donors, processed, frozen and stored under an MHRA manufacturing licence held at the University of Birmingham Microbiome Treatment Centre. Thawed FMT samples will be administered to patients either via 8 naso-gastric infusions given over 4 days starting on the day of randomisation, and then again for 4 days in week 4 for foregut delivery (total of 240g of stool) or via one colonoscopic infusion followed by 7 weekly enemas according to the hindgut protocol (total of 360g of stool). Patients will be followed up weekly for 8 weeks, and then at 12 weeks. The aims of this pilot study are i) to determine which FMT administration route (naso-gastric or colon) should be investigated in a randomised double-blind, placebo-controlled trial; and ii) to determine if a full randomised controlled trial is feasible. The primary outcome will be a composite assessment of both qualitative and quantitative data based on efficacy (clinical response), acceptability and safety. At the end of the pilot, decisions will be made regarding the feasibility of a full randomised double-blind, placebo-controlled trial and, if deemed feasible, which route of administration should be used in such a study.

Ethics and dissemination

Ethical approved for this study has been obtained from the East Midlands-Nottingham Research Ethics Committee (REC 17/EM/0274). At the end of the study, findings will be reported at national and international gastroenterology meetings and published in peer-reviewed journals.

Strengths and limitations of this study

<u>Strengths</u>

- Matching of single donor with each patient - this will allow detailed exploration of donor characteristics associated with a favourable patient response

For peer review only - http://bmjopen.bmj.com/site/about/guidelines.xhtml

For peer review only

- Intensive FMT treatment regimen – shown to be a major contributing factor in clinical response to FMT
- Assessment of mechanistic outcomes and donor diet – this will help to understand mechanisms associated of response to FMT.
- Qualitative data from clinicians and patients informing the potential future randomised controlled trial.

Limitations

- This study is limited to assessing the feasibility of a randomised controlled trial and the best route of FMT delivery in UC patients. The assessment of the efficacy of FMT in achieving and maintaining remission for patients with UC needs to be tested in a future full-scale randomised controlled trial.

Trial registration number ISRCTN74072945

Registered with: International Standard Registered Clinical Study Number (ISRCTN).

Data set: 74072945

Protocol version: 4.0 17-Aug-2018

Funder: Efficacy and Mechanism Evaluation - NIHR

Sponsor: University of Birmingham

Keywords: Faecal Microbiota Transplant, Ulcerative Colitis, Randomised Controlled Pilot Study

Word Count of manuscript: 5333

For peer review only - http://bmjopen.bmj.com/site/about/guidelines.xhtml

INTRODUCTION

In the inflammatory bowel diseases (IBD), Crohn's disease (CD) and ulcerative colitis (UC), there is imbalance or 'dysbiosis' of the gut microbiota compared to the healthy bowel, with individuals having IBD showing reduced bacterial diversity compared to healthy individuals.[1,2]Indeed, the currently accepted paradigm concerning the pathogenesis of IBD involves an aberrant immunological response to the intestinal microbiota in a genetically susceptible host.[3] Whether the observed dysbiosis represents "cause" or "effect" remains unanswered. Faecal Microbiota Transplant (FMT) is the infusion of a faecal suspension from a healthy donor into the gastrointestinal tract of a patient with disease, and there is interest in the potential of this technique for modifying the gut microbiome as a potential treatment for IBD.[4]

Since the first descriptions of CD and UC at the beginning of the twentieth century, it has been strongly suspected that the gut microbiota may have a defining role in the pathogenesis of IBD.[5] Early culture-based studies underestimated the complexity of the microbiota, and were hampered by various culture biases and the challenge of enumerating fastidious bacteria with complex growth conditions. With the advent of cheap high-throughput genetic sequencing techniques allied with complex bioinformatics capability, there has been a revolution in our understanding of the composition and function of the colonic microbiome. As a result of studies on the microbiome both in patients with IBD and animal models, we know that patients with IBD (either CD or UC) have, at the phylum level, a reduction in Firmicutes and a relative increase in Proteobacteria.[2,6]

Accumulating data suggests that alteration in the gut microbiome plays a central role in driving UC; datasets highlighting the importance of *Roseburia hominis*[7], *Faecalibacterium prausnitzii*[8] and *Akkermansia muciniphila*[9] in anti-inflammatory responses in UC have been published. Attempts to alter the microbiome with probiotics, whilst disappointing in CD, have shown some promise in UC.[10,11]Since the first exploratory use of FMT to treat UC in 1989[12], numerous case series have been published demonstrating encouraging efficacy signals.[13]This has led to investigators testing FMT as a treatment for UC and to date there have been four randomised controlled trials (RCTs) of FMT for the treatment of UC.[14] The first was a trial of fresh and frozen enemas which were delivered weekly for 6 weeks, water being used as placebo.[15] There was a significant increase in remission at the primary endpoint in the active versus placebo arm. In the second study, fresh FMT by naso-jejunal delivery was used and autologous stool was used as placebo.[16] Although the results favoured active treatment, they were not statistically significant. The last two RCTs were reported in 2017[17,18]and both used

For peer review only - http://bmjopen.bmj.com/site/about/guidelines.xhtml

frozen FMT from pooled donors compared to saline placebo and involved intensive treatment regimens. Both achieved statistically significant results in favour of active treatment, with remission rates comparing active and placebo of 32% versus 9%[18] and 27% versus 8% respectively[17].

As a result of this recent work, there is great interest in FMT as a possible treatment for UC, but the optimal route of delivery remains unknown. Before we can undertake a full-scale double-blind RCT of FMT versus placebo, we need to undertake a pilot study to determine the optimum route of FMT delivery and assess the feasibility (recruitment, protocol adherence) of undertaking a trial of FMT in UC. In this pilot study, we will compare naso-gastric (NG) versus colonoscopic (COLON) delivery of FMT, using an intensive treatment regimen, in patients with active UC to investigate the optimum route of FMT delivery for the treatment of UC. We will assess the effectiveness and acceptability of the two routes of FMT delivery, and whether FMT by either NG or COLON is suitable to take forward to a full RCT against placebo, and whether such a trial is feasible. Here we describe the protocol for the STOP-COLITIS pilot study.

For peer review only

For peer review only - http://bmjopen.bmj.com/site/about/guidelines.xhtml

METHODS AND ANALYSIS

Study design

A prospective, multi-centre open-label, randomised pilot study to assess two possible routes of FMT delivery for the treatment of UC. Thirty patients with active UC will be randomised to receive FMT via either the naso-gastric route or by direct delivery to the colon. Patients will be recruited from three hospitals in the United Kingdom (Queen Elizabeth Hospital, Birmingham; St Mark's Hospital, London; and Glasgow Royal Infirmary).

At the end of the pilot, the data will be reviewed by an Independent Oversight Committee (IOC), consisting of two consultant gastroenterologists and a professor of medical statistics and clinical trials, who, according to the pre-specified STOP/GO criteria, (see below) will decide (1) which route of FMT administration (NG or COLON) is most appropriate to investigate in the full randomised double-blind, placebo-controlled trial, and (2) whether it is feasible to proceed to the main randomised controlled trial.

Sample size

As this is a pilot study, no formal sample size calculation was undertaken as the study is not designed or powered to detect a statistically significant difference in efficacy between the two FMT methods of delivery. The recruitment target for this study is 30 patients.

Aims and objectives

The aims of this pilot study are:

i) to determine which FMT administration route (NG or COLON) should be investigated in a randomised double-blind, placebo-controlled trial; and

ii) to determine if a full randomised controlled trial is feasible.

In order to achieve these aims, the pilot study has the following clinical (quantitative), qualitative and mechanistic objectives.

Clinical objectives

To assess:

- Whether FMT by the NG route induces clinical response in patients with active UC;
- Whether FMT by the colonic route induces clinical response in patients with active UC;
- Tolerability and safety;

For peer review only - http://bmjopen.bmj.com/site/about/guidelines.xhtml

- Which route of FMT delivery (if any) is suitable to investigate in the RCT.

Qualitative objectives

To assess:

- Patient acceptability of FMT (NG);
- Patient acceptability of FMT (COLON).

Mechanistic objectives

To assess:

- Whether FMT by either route is associated with a change in faecal calprotectin as a surrogate marker of colonic inflammation;
- Changes in the colonic microbiome and metabolome (short chain fatty acids (SCFA)) induced by FMT via each route;
- Reduction in C-reactive protein (CRP);

Other objectives

- Effect of diet (donors) on microbiome;
- Effect of time from stool donation to treatment on success of FMT.

Outcome measures

The primary outcome will be a composite assessment of both qualitative and quantitative data relating to efficacy, acceptability, safety and mechanistic outcomes.

Clinical outcome measures

- Clinical response (primary measure of efficacy) defined as ≥3 point reduction in the full Mayo score from randomisation to week 8, and 30% reduction from randomisation and at least 1 point reduction of rectal bleeding sub-score or an absolute rectal bleeding sub-score of 0 or 1;
- Time to clinical response (where clinical response is defined as ≥2 point reduction in partial Mayo);
- Clinical remission at week 8 (full Mayo score of ≤2, with no sub-score >1);
- Participant's weight at week 8 and week 12;
- Quality of Life (QoL) using the generic Short-Form 36 (SF-36) and the disease specific Inflammatory Bowel Disease Questionnaire (IBDQ) at week 8 and week 12;
- Adherence to FMT;

For peer review only - http://bmjopen.bmj.com/site/about/guidelines.xhtml

- Adverse events (AE) and serious adverse events (SAE).

Qualitative data

Patient acceptability will be assessed through qualitative research interviews. During these interviews, patients from both groups will be asked about their perspectives and experience of FMT during the pilot trial, including receiving the intervention, recovering from the intervention, and their symptoms and quality of life. Further examples of interview questions can be found in the Supplementary file 1.

Mechanistic outcome measures

- Faecal calprotectin;
- Measures of microbiome (faecal and mucosal) - specifically shifts in alpha diversity following FMT
- Mucosal healing;
- Urinary metabolome (SCFA);
- CRP;

Other outcome measures

- Association between the donors' and recipients' dietary profile and microbiome;
- Time, i.e. number of days, from donor stool processing to treatment for association between efficacy and freezer life of FMT.

Donor sample acquisition and processing

Donors will be recruited following advertisement from healthy un-related anonymous individuals living in Birmingham. We are excluding healthcare workers due to their potential exposure to microbes affecting the microbiome. Donors must be ≥18 and <50 years of age, have a normal morning bowel habit, have a normal body mass index (BMI; ≥18.5 and ≤25), be non-smokers (not smoked for at least 12 months) and have no recent history of diarrhoea or rectal bleeding. Potential donors will undergo rigorous screening using a health-screening questionnaire. Those who are eligible following this screening will be consented for the donation process and have blood and faecal samples taken to test for transmissible pathogens in accordance with UK, AGA and European guidelines.[19–21]Individuals who pass the screening process will be invited to donate morning faecal samples for 10 days and to deliver these for processing within 6 hours of defaecation. At first donation, they will also be asked to complete the EPIC-Norfolk food frequency questionnaire.[22]

For peer review only - http://bmjopen.bmj.com/site/about/guidelines.xhtml

Donations will be processed and stored under an MHRA manufacturing licence at the University of Birmingham Microbiome Treatment Centre. Stool collection and processing were performed under aerobic conditions. Donations will be prepared in saline with 10% glycerol added as a cytoprotectant. Samples will then be stored frozen at -80°C for up to 24 weeks. Samples will be dispatched for use at each site as required. An aliquot from each donated sample will be kept for analysis in the event of adverse events occurring as a result of FMT and samples from each donation period will be sent for genetic sequencing and metabolic analysis. At the end of each 10 day donation period, donors will receive financial compensation and will undergo an exit health questionnaire and be asked to provide a further stool sample to test for pathogens. Further information on the donor sample processing is in the protocol and a lab SOP for donor preparation (available on request).

Participants

Inclusion criteria

- Adult patients (aged between 16 and 70 years) with clinically confirmed UC for at least 12 weeks prior to the screening visit;
- Partial Mayo score of ≥4 and ≤8 despite stable disease maintenance treatment with 5-aminosalicylates with or without immunomodulators, or on no treatment;
- Rectal bleeding subscore of ≥1 on the partial Mayo;
- Able to give written, signed informed consent.

Exclusion criteria

- Stool positive for *Clostridium difficile* or infection by either PCR or ELISA;
- Positive for Hepatitis A/B/C, and/or Human Immunodeficiency Virus (HIV) infection;
- Antibiotics in the preceding 12 weeks prior to date of the screening visit;
- Systemic/topical steroids in the preceding 2 weeks prior to the date of the screening visit;
- Biologics in the preceding 12 weeks prior to the date of screening visit;
- Commercial probiotics and prebiotics in the preceding 12 weeks prior to the date of the screening visit;
- On oral nutritional supplements or enteral/parenteral nutrition in the preceding 4 weeks prior to the date of the screening visit;
- Pregnant or lactating;
- Not willing to take appropriate contraceptive measures to prevent pregnancy during trial participation.

For peer review only - http://bmjopen.bmj.com/site/about/guidelines.xhtml

Participant enrolment

Potentially eligible patients who express an interest in participating in the trial will be consented via a two-stage consent process. Participant information sheets will be provided to facilitate the consent process.

Registration and screening visit

The first stage will involve consent for trial-specific screening activities, and consent to collect stool and urine samples for the mechanistic sub-studies. Patients will undergo basic physiological assessments (pulse, blood pressure, temperature, height and weight) and baseline blood tests. They will be provided with a diary to record bowel symptoms (so that the partial Mayo score can be calculated at the randomisation visit), stool sample kits and bowel preparation kits (Moviprep®). They will be asked to return the *C. difficile* stool sample as soon as possible, so that the result is available prior to the randomisation visit.

Following the screening visit, the qualitative researcher will arrange for an interview with the patient to take place prior to their randomisation visit (see below). The research team at the hospital will contact the patient to notify them of their stool result. If they tested negative for *C.difficile*, they will be invited to attend the randomisation visit. Instructions will be given on when to take the bowel preparation, and they will be asked to collect a stool sample on the same day prior to taking the bowel preparation, which they will bring to the randomisation visit.

Randomisation visit

The second stage is consent for entry into the trial. Following confirmation of all eligibility criteria and consent to randomisation, basic physiological assessments will be undertaken, blood test results will be checked, a urine sample will be taken for pregnancy testing in women and metabolomics and the partial Mayo score will be calculated from diaries. The patient will be asked to complete the baseline QoL questionnaires; IBDQ and SF-36. All patients will have a colonoscopy to assess disease (done after randomisation), so that a full Mayo score can be calculated, and to collect mucosal biopsies.

Randomisation

Patients will be randomised at the level of the individual in a 1:1 ratio to either NG or COLON delivery of FMT. Randomisation will be provided by a computer-generated program at the Birmingham Clinical Trials Unit (BCTU). A minimisation algorithm will be used to ensure balance in the treatment allocation over the following variables:

For peer review only - http://bmjopen.bmj.com/site/about/guidelines.xhtml

- Partial Mayo score (4-5 or 6-8);
- Current smoking status (current smoker: yes or no (not smoked for the past 12 months));

A 'random element' will be included in the minimisation algorithm, so that each patient has a probability (unspecified here), of being randomised to the opposite treatment that they would have otherwise received.

Interventions

Before the initial FMT treatment, all patients will receive standard bowel preparation: 2 litres of reconstituted Moviprep® solution within the 24 hours prior to procedure.

Naso-gastric treatment (NG)

Patients randomised to NG treatment will be pre-treated with a proton pump inhibitor (lansoprazole 30mg) and a prokinetic agent (domperidone 10mg) at least 30 minutes prior to each FMT infusion to reduce gastric acid secretion and prevent the risk of gastro-oesophageal regurgitation. Naso-gastric tubes (NGT) will be positioned and checked for correct position as per local protocol. Following thawing at room temperature, 50ml thawed FMT containing 30g faeces will be infused. Following the first treatment, patients will return for the next three days for further treatment following an overnight fast. It will be the individual's choice whether they wish to retain the NGT for the 4 day treatment course or have the tube removed and re-passed every day. On the last day of treatment, the NGT will be removed to be replaced for the second course of treatment. At week 4, patients will return for a further 4 FMT infusions over 4 consecutive days following a fast from midnight.

In summary, patients will receive 30g FMT in 50ml aliquots for NG administration each day for 4 days at the start of the trial (starting on the day of randomisation), and then again for 4 days in week 4 (total FMT dose 240g).

Colonoscopic treatment (COLON)

Patients randomised to COLON treatment will receive a thawed 250ml aliquot of FMT (containing 150g faeces) on the day of randomisation. This will be delivered to the colon using a spray catheter, with 125ml sprayed into the caecum to treat the right side of the bowel and the remaining 125ml sprayed directly onto the rest of the colon. Following treatment, patients will receive a single dose of loperamide. Patients will return on a weekly basis to receive 100ml enema up to week 7 containing 50ml of saline and 30g faeces, followed by loperamide each time.

For peer review only - http://bmjopen.bmj.com/site/about/guidelines.xhtml

In summary, patients will receive 150g of stool in 250ml for colonic administration on the day of randomisation followed by 30g of stool in 100ml in normal saline for administration by enema for 7 weeks (total FMT dose 360g).

Data Collection

All patients will be followed weekly up to week 8, and then again at week 12. At each visit, the partial Mayo score will be calculated from the patient diaries, and medication use and any adverse events occurring will be recorded. Stool samples will be collected at weeks 2, 4, 6, 8 and 12, and blood samples will be taken for CRP assessment at weeks 4, 6 and 8. At weeks 8 and 12, physiological data, a urine sample and the SF-36 and IBDQ QoL questionnaires will also be collected. At week 8, a flexible sigmoidoscopy will be undertaken to calculate the full Mayo Score. See supplementary figure 1.

Timelines

We completed recruitment into this pilot study in April 2019. Data collection will be completed by July 2019, and analysis of the data will commence in August 2019.

Supplementary figure 1 - Trial schema and data collection outline

Qualitative Research: Patient and clinician experience and acceptability of FMT and trial processes

A comparison of the acceptability of the two routes of FMT being used in this study is key to the outcome of this pilot study. A qualitative researcher will conduct interviews with patients participating in both arms of the trial. Each patient will take part in a semi-structured interview at two time points; following the screening visit prior to randomisation and after the 12-week follow up visit. These one-to-one interviews will take place either in person, by telephone or Skype, as preferred by the patients. The initial interview will focus on participants' experience of UC and treatment to date, along with their understanding and expectations for FMT and the pilot study. A background questionnaire collecting socio-demographic and clinical details will also be administered. During the follow up interview, patients' actual experience within the pilot study, including that of FMT (by NG and COLON) and of related trial processes and procedures will be explored. Interviews will also gather data on patient perspectives regarding important impacts and outcomes of FMT, thus providing the

For peer review only - http://bmjopen.bmj.com/site/about/guidelines.xhtml

opportunity to compare these with the outcome measures selected for inclusion in the pilot and (later) full RCT. Further interviews with patients who withdraw from FMT treatment early and with a small sample of patients who decline consent to participate following screening (should this happen), will provide in-depth understanding of these decisions. Interviews will also be conducted with staff at the pilot sites to review their experience of trial conduct e.g. the acceptability and logistics of complex trial procedures within clinical environments. Interviews schedules can be found in Supplementary file 1.

Data from this qualitative research will contribute to an assessment of patient and clinician experience and acceptability of FMT within the pilot context, and along with recruitment and drop-out rates, and data regarding clinical response, this will inform a decision about whether the NG or COLON route of delivery is taken forward to the full RCT. The qualitative data will also contribute to the refinement and optimisation of trial processes prior to the full RCT.

Analysis of qualitative data

Interviews will be recorded with the consent of participants and transcribed clean verbatim for analysis. Analysis will be conducted with reference to recordings, transcripts and field notes taken at the time of data collection. A thematic analysis of content will be informed by the Framework analytical approach.[23] Following initial familiarisation with the interview data, development of thematic frameworks and data coding will proceed in an iterative manner. Data collection and analysis will run concurrently so that emergent analytical themes can inform further data collection, and in particular comparative analytical questioning between patients allocated to NG or COLON.

Mechanistic studies

There will be two aspects to mechanistic studies undertaken as part of this trial; non-invasive assessment of UC activity and assessment of patient's microbiome response to FMT in comparison with that of donor microbiome at baseline. The study will also investigate donor diet with regard to baseline microbiome and treatment effect.

Non-invasive assessment of UC activity

This will be undertaken using the serum biomarker CRP and faecal calprotectin which will be measured using a standard protocol at one central laboratory in Glasgow.

Microbiome related assays

For peer review only - http://bmjopen.bmj.com/site/about/guidelines.xhtml

16S ribosomal DNA sequencing and metagenomics

16S ribosomal DNA sequencing will be performed on all samples collected during the study: donor faeces (fresh and frozen from day 1 and day 10 donations), faeces from the recipient at baseline, weeks 2, 4, 6, 8, and 12 and mucosal biopsies taken from the right colon, left colon and rectum at baseline and from left colon and rectum at week 8. Together these samples will be compared to clinical data, and correlations found with faecal calprotectin, in order to find the samples most likely to be biologically informative as a marker of disease remission. Additionally, and based on the results of the initial 16S analysis, a shotgun metagenomics approach will be undertaken on around 200 selected samples to give insights regarding functional enrichment. For a certain subset of samples where additional strain resolution is required (e.g. recovery of complete genomes of interest) or linkage between extra-chromosomal elements such as plasmids and phages, further samples will be subject to long-read sequencing.

Major bacterial metabolites and metabolomics on faecal and urine samples

Major bacterial metabolites including faecal short and medium chain fatty acids previously implicated in the aetiology and mucosal inflammation of IBD will be measured using gas chromatography and other assays as described previously.[24–26] Analysis of short chain fatty acids in the samples will be carried out on faecal samples from the donors on day 1 and day 10 and on patients at baseline, week 8 and week 12.

Donor assessment of dietary intake

The habitual dietary pattern of the donors will be assessed using the validated food frequency questionnaire as used in the EPIC-Norfolk study in the UK.[27] Data from the EPIC questionnaires will be transferred to Glasgow for analysis. Energy, macronutrient and fibre intake will be estimated and expressed in nutrient ranks and quartiles and will also be compared against Department of Health recommendations and UK National Diet and Nutrition Survey results.

Analysis of outcome measures

The primary outcome from the pilot study will be a recommendation about which route of administration (NG or COLON) to take forward to the main RCT, and whether a full RCT is feasible. The decision will be based on assessing the two methods of delivery using a composite assessment of both qualitative and quantitative data on efficacy using clinical response, acceptability and safety.

For peer review only - http://bmjopen.bmj.com/site/about/guidelines.xhtml

For peer review only

The primary comparison will comprise of those randomised to FMT by the NG route versus those randomised to FMT by the COLON route. All analyses will be based on the intention to treat principle. Data will be presented as summary statistics and differences between groups will be presented with 95% confidence intervals (CI) from 2-sided tests. No formal hypothesis testing will be undertaken and no p-values will be presented.

Primary outcome measure of efficacy for STOP/GO

The primary measure of efficacy for the STOP/GO is clinical response at week 8. The number and percentage of patients achieving a clinical response in the two treatment groups will be reported along with the 95% CI. A log-binomial model will be fitted to obtain a relative risk and 95% CI, adjusting for the minimisation variable smoking status and baseline full Mayo score.

Secondary Clinical Outcomes Measures for STOP/GO

The number and percentage of patients achieving clinical remission in the two treatment groups will be reported. A log-binomial model will be fitted to obtain a relative risk and 95% CI, adjusting for the minimisation variables: smoking status and baseline partial Mayo score. Time to clinical response (based on the partial Mayo score) will be presented graphically as a Kaplan-Meier plot. A Cox proportional hazards regression model will be fitted to obtain a hazard ratio and 95% CI. Continuous data (e.g. weight, QoL scores) will be summarised using means and standard deviations presented at each time point (baseline, week 8 and week 12). The week 8 and week 12 data will be compared by treatment group using linear regression models with the minimisation variables and baseline values included in the model, with the mean differences and 95% CI presented.

Tolerability will be assessed quantitatively using adherence. A patient will be considered adherent if they receive at least 70% of their intended FMT dose. The number and percentage of patients who are deemed adherent to the FMT treatment in the two treatment groups will be reported along with the 95% CIs.

Adverse event and SAE data will be tabulated. For each treatment group, the number of adverse events and the number and percentage of patients experiencing an adverse event will be reported. Data on SAEs will be reported in the same way.

STOP/GO guidelines

For peer review only - http://bmjopen.bmj.com/site/about/guidelines.xhtml

At the end of the pilot, the IOC will be convened to review the data. This group will make a recommendation to the Trial Management Group on (1) which route (if any) of FMT is appropriate to take forward to the full trial and (2) whether it is feasible to proceed to the full RCT.

To determine whether we will proceed forward to the main RCT, a pragmatic review of the pilot data in terms of assessing treatment efficacy (based on clinical response), tolerability, and patient acceptability will be undertaken. STOP/GO guidelines will be used to determine whether to proceed forward to the main RCT, and we propose then to take forward the preferred method of FMT delivery into a randomised double-blind placebo-controlled trial with a clinical efficacy outcome (clinical remission).

The first STOP/GO decision will be on which route of FMT to take forward to the full RCT. The pilot is not powered to show differences in the two modes of delivery (NG or COLON); therefore a decision or recommendation on which route to use in the main trial requires expert judgement and cannot be made based on a purely numerical process. The following data will be reviewed by the IOC:

- The proportion of patients who achieve a clinical response following FMT by the NG route†;
- The proportion of patients who achieve a clinical response following FMT by the COLON†;
- Whether FMT by either route is associated with a change in faecal calprotectin;
- Whether FMT by either route is associated with changes in the colonic microbiome and metabolome;
- Tolerability and safety for each route;
- Patient acceptability of FMT by the NG route through the qualitative interviews (including advantages and disadvantages of the NG route);
- Patient acceptability of FMT by the COLON route through the qualitative interviews (including advantages and disadvantages of the COLON route).

† A threshold of achieving a clinical response in around 40% of patients treated may be used. This threshold will be agreed after discussions by the Trial Management Group and the IOC prior to any data analysis being performed.

Following review of the above data, whether the IOC can recommend a route to take forward to the main trial, will form the basis of the first STOP/GO decision.

a) IOC unable to recommend one particular route as neither route felt to be satisfactory. DECISION: Do not proceed to main RCT.

For peer review only - http://bmjopen.bmj.com/site/about/guidelines.xhtml

b) IOC recommends a route of FMT delivery for use in the main RCT. DECISION: Proceed to STOP/GO decision stage 2 of pilot.

Once the route of FMT delivery has been selected, a second STOP/GO assessing feasibility will be used to determine whether to proceed to the main RCT. This will be based on the following:

1) That the IOC are able to recommend a route.

2) That the recruitment of the 30 patients in the pilot averages 0.7 patients per week in each open site.

3) That 10 of the 15 patients in the route cohort selected for the main study received at least 70% of their intended FMT dose. For example, 70% of the NG route (240g) is 168g, which requires the patient to receive at least 6 infusions (30g; 6 out of the 8 FMT infusions i.e. patient can miss no more than two doses); and 70% of the COLON route (360g) is 252g, which requires the patient to receive the first infusion by colonoscopy (150g) and then at least 4 out of 7 of the enema infusions (30g) i.e. patient can miss no more than three doses.

4) That the IOC have not identified any safety concerns.

Following review of the above data, whether the IOC are able to recommend that a full RCT is feasible will form the basis of the second STOP/GO decision.

a) IOC consider a RCT unfeasible. DECISION: Do not proceed to main RCT.

b) IOC consider a RCT feasible. DECISION: Provide report to funder with recommendation of IOC, in order for a final decision to be made on progression to the full RCT.

Patient and Public Involvement

Our protocol was developed in consultation with a research PPI group referred to as the Clinical Research Ambassador Group (CRAG), based at University Hospital NHS Foundation Trust. One of the co-applicants is a patient with IBD and also Chairperson for the West Midlands Group of Crohn's and Colitis UK (CCUK) and she helped develop the proposal. The PPI has strong links with CCUK and this will significantly aid dissemination of findings nationally to patients, relatives and health professionals. As we are aware that acceptability of FMT in UC has not been extensively studied, we designed the pilot study to investigate patient acceptability of the therapy by regular questionnaires, individual interviews and group discussions. We conducted a survey of our own IBD patients from the outpatient department at University Hospital Birmingham. Of the 74 patient surveyed the vast majority of

For peer review only - http://bmjopen.bmj.com/site/about/guidelines.xhtml

patients would accept FMT as a treatment for IBD and over 80% would consider involvement in a trial of FMT in IBD. Qualitative interviews with patients will be conducted at baseline and at the end of the study and these findings will feed into the discussions regarding progression to a full-scale RCT. Patients recruited to the study will not necessarily directly assist with the recruitment of other patients.

Ethics And Dissemination

The trial will be performed in accordance with the recommendations guiding physicians in biomedical research involving human subjects, adopted by the 18th World Medical Association General Assembly, Helsinki, Finland, June 1964, amended at the 48th World Medical Association General Assembly, Somerset West, Republic of South Africa, October 1996.

The trial will be conducted in accordance with the Research Governance Framework for Health and Social Care, the applicable UK Statutory Instruments (which include the Medicines for Human Use Clinical Trials 2004 and subsequent amendments and the General Data Protection Regulation (GDPR) (EU) 2016/679 and Human Tissue Act 2008) and Guidelines for Good Clinical Practice (GCP). This trial will be carried out under a Clinical Trial Authorisation in accordance with the Medicines for Human Use Clinical Trials regulations.

Ethical approved for this study has been obtained from the East Midlands-Nottingham Research Ethics Committee (REC 17/EM/0274).

Publication policy

At the end of the study, a meeting will be held to allow discussion of the main results among the collaborators prior to publication. The success of the study will depend entirely on the wholehearted collaboration of a large number of doctors, nurses and others. For this reason, the chief credit for the main results will be given not only to the central supervisory committees and/or organisers, but to all those who have collaborated in the trial.

The findings will be reported at national and international Gastroenterology meetings and published in peer-reviewed journals.

Authors' Contributions

For peer review only

MNQ, MY, JS, CB, NS, PH, VLM, AH, DG, RH, NI, LM, SL, CH, NC, and TI were all involved in developing the clinical protocol and authoring the manuscript , with MNQ being the lead author, TI is the Chief Investigator. NI is the senior statistician on the project. She was involved in the trial design, protocol development and led the development of the proposed statistical analyses. CAH is the trial statistician and was involved in protocol development and produced the statistical analysis plan for this study. SL is the trial co-ordinator and with LM developed the trial management and governance aspects of the manuscript. KG, NL, CQ contributed the mechanistics and bio-informatics expertise. JM and CM authored the qualitative research section of the manuscript. We thank our patient advisor NC for her help with developing this protocol. We used the SPIRIT checklist when writing this protocol.

Funding Statement

This study is funded by the National Institute for Health Research (NIHR) as part of the Efficacy and Mechanism Evaluation (EME) programme.

Competing Interests Statement

None

For peer review only - http://bmjopen.bmj.com/site/about/guidelines.xhtml

For peer review only

REFERENCES

1. Qin, J. *et al.* A human gut microbial gene catalogue established by metagenomic sequencing. *Nature* **464**, 59 (2010).
2. Gerasimidis, K. *et al.* Decline in presumptively protective gut bacterial species and metabolites are paradoxically associated with disease improvement in pediatric Crohn's disease during enteral nutrition. *Inflamm. Bowel Dis.* **20**, 861–871 (2014).
3. de Souza, H. S. P. & Fiocchi, C. Immunopathogenesis of IBD: current state of the art. *Nat. Rev. Gastroenterol. Hepatol.* **13**, 13–27 (2016).
4. Anderson, J. L., Edney, R. J. & Whelan, K. Systematic review: faecal microbiota transplantation in the management of inflammatory bowel disease. *Aliment. Pharmacol. Ther.* **36**, 503–516 (2012).
5. Sartor, R. B. Microbial influences in inflammatory bowel diseases. *Gastroenterology* **134**, 577–594 (2008).
6. Kostic, A. D., Xavier, R. J. & Gevers, D. The microbiome in inflammatory bowel disease: current status and the future ahead. *Gastroenterology* **146**, 1489–1499 (2014).
7. Machiels, K. *et al.* A decrease of the butyrate-producing species Roseburia hominis and Faecalibacterium prausnitzii defines dysbiosis in patients with ulcerative colitis. *Gut* **63**, 1275–1283 (2014).
8. Varela, E. *et al.* Colonisation by Faecalibacterium prausnitzii and maintenance of clinical remission in patients with ulcerative colitis. *Aliment. Pharmacol. Ther.* **38**, 151–161 (2013).
9. Manichanh, C., Borruel, N., Casellas, F. & Guarner, F. The gut microbiota in IBD. *Nat. Rev. Gastroenterol. Hepatol.* **9**, 599–608 (2012).
10. Sood, A. *et al.* The probiotic preparation, VSL#3 induces remission in patients with mild-to-moderately active ulcerative colitis. *Clin. Gastroenterol. Hepatol. Off. Clin. Pract. J. Am. Gastroenterol. Assoc.* **7**, 1202–1209, 1209.e1 (2009).

For peer review only

For peer review only - http://bmjopen.bmj.com/site/about/guidelines.xhtml

11. Tursi, A. *et al.* Treatment of Relapsing Mild-to-Moderate Ulcerative Colitis With the Probiotic VSL#3 as Adjunctive to a Standard Pharmaceutical Treatment: A Double-Blind, Randomized, Placebo-Controlled Study. *Am. J. Gastroenterol.* **105**, 2218 (2010).
12. Bennet, J. D. & Brinkman, M. Treatment of ulcerative colitis by implantation of normal colonic flora. *Lancet Lond. Engl.* **1**, 164 (1989).
13. Costello, S. P. *et al.* Systematic review with meta-analysis: faecal microbiota transplantation for the induction of remission for active ulcerative colitis. *Aliment. Pharmacol. Ther.* **46**, 213–224 (2017).
14. Paramsothy, S. *et al.* Faecal Microbiota Transplantation for Inflammatory Bowel Disease: A Systematic Review and Meta-analysis. *J. Crohns Colitis* **11**, 1180–1199 (2017).
15. Moayyedi, P. *et al.* Fecal Microbiota Transplantation Induces Remission in Patients With Active Ulcerative Colitis in a Randomized Controlled Trial. *Gastroenterology* **149**, 102-109.e6 (2015).
16. Rossen, N. G. *et al.* Findings From a Randomized Controlled Trial of Fecal Transplantation for Patients With Ulcerative Colitis. *Gastroenterology* **149**, 110-118.e4 (2015).
17. Paramsothy, S. *et al.* Multidonor intensive faecal microbiota transplantation for active ulcerative colitis: a randomised placebo-controlled trial. *Lancet Lond. Engl.* **389**, 1218–1228 (2017).
18. Costello, S. P. *et al.* Effect of Fecal Microbiota Transplantation on 8-Week Remission in Patients With Ulcerative Colitis: A Randomized Clinical Trial. *JAMA* **321**, 156–164 (2019).
19. Letter from the Presidents of the Infectious Disease Society of America, the American Gastroenterological Association, the American Society for Gastrointestinal Endoscopy et al., Current Consensus Guidance on Donor Screening and Stool Testing for FMT, July 5, 2013, http://www.gastro.org/research/Joint_Society_FMT_Guidance.pdf (accessed December, 2017).
20. Cammarota, G. *et al.* European consensus conference on faecal microbiota transplantation in clinical practice. *Gut* gutjnl-2016-313017 (2017). doi:10.1136/gutjnl-2016-313017
21. Mullish, B. H. *et al.* The use of faecal microbiota transplant as treatment for recurrent or refractory Clostridium difficile infection and other potential indications: joint British Society of

For peer review only - http://bmjopen.bmj.com/site/about/guidelines.xhtml

Gastroenterology (BSG) and Healthcare Infection Society (HIS) guidelines. *Gut* **67**, 1920–1941 (2018).

22. Moskal, A. *et al.* Nutrient Patterns and Their Food Sources in an International Study Setting: Report from the EPIC Study. *PLoS ONE* **9**, (2014).
23. *Ritchie, J., Spencer, L. & O'Connor, W. Carrying out qualitative analysis in: Qualitative Research Practice. J Ritchie and L Lewis (eds) London: Sage (2003).*
24. Kamleh, A. *et al.* Metabolomic profiling using Orbitrap Fourier transform mass spectrometry with hydrophilic interaction chromatography: a method with wide applicability to analysis of biomolecules. *Rapid Commun. Mass Spectrom. RCM* **22**, 1912–1918 (2008).
25. Creek, D. J., Jankevics, A., Burgess, K. E. V., Breitling, R. & Barrett, M. P. IDEOM: an Excel interface for analysis of LC-MS-based metabolomics data. *Bioinforma. Oxf. Engl.* **28**, 1048–1049 (2012).
26. Creek, D. J. *et al.* Toward global metabolomics analysis with hydrophilic interaction liquid chromatography-mass spectrometry: improved metabolite identification by retention time prediction. *Anal. Chem.* **83**, 8703–8710 (2011).
27. Welch, A. A., Luben, R., Khaw, K. T. & Bingham, S. A. The CAFE computer program for nutritional analysis of the EPIC-Norfolk food frequency questionnaire and identification of extreme nutrient values. *J. Hum. Nutr. Diet. Off. J. Br. Diet. Assoc.* **18**, 99–116 (2005).

For peer review only

For peer review only - http://bmjopen.bmj.com/site/about/guidelines.xhtml

Supplementary Figure 1 – Trial Schema

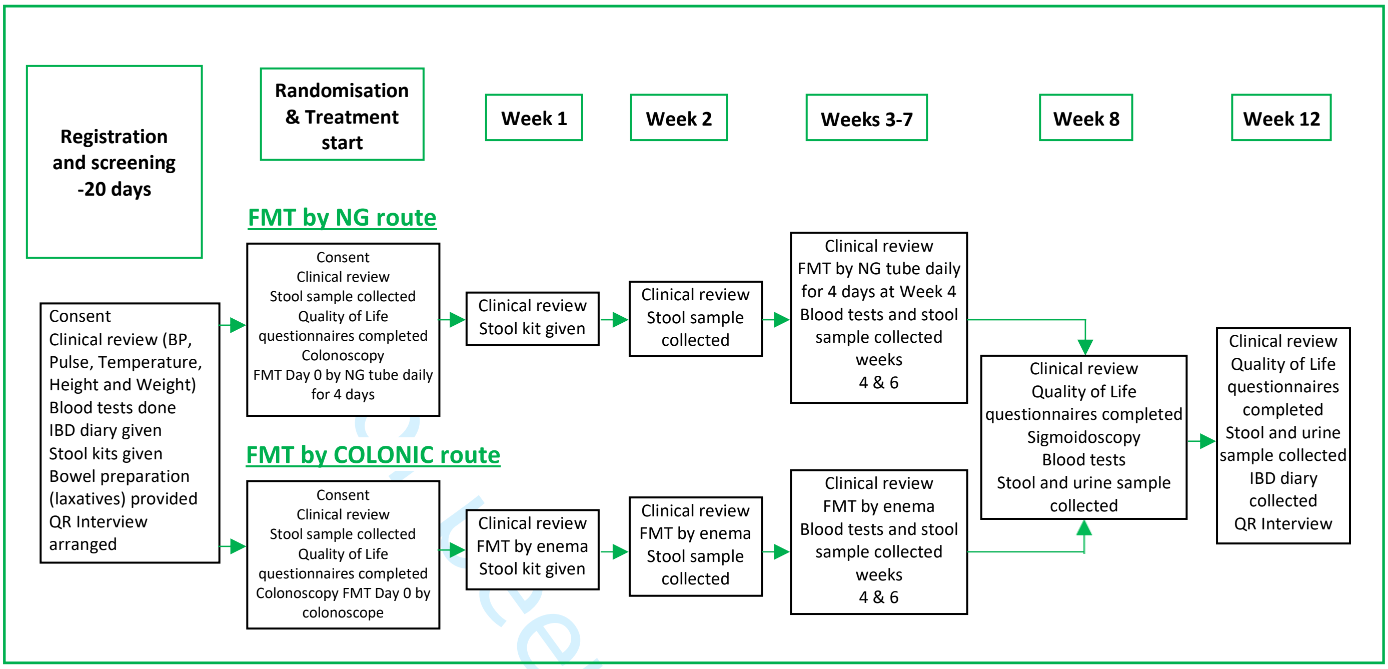

For peer review only - http://bmjopen.bmj.com/site/about/guidelines.xhtml

INTERVIEW SCHEDULE STOP-COLITIS T1 (Baseline) - PATIENTS

INTRODUCTION

Thank the participant for agreeing to take part.

Ensure that the consent form has been signed. Keep a copy for the site file and give a copy back to the participant.

Statement on confidentiality, right to withdraw consent, recording of the interview.

Review the purpose of the study in general.

Emphasize the value of their views and opinions – there are no right or wrong answers.

Ask if the participant have any questions before starting the interview.

ICEBREAKER

Background, interviewee details and icebreaker e.g. explore interviewee background, have family, and why they were interested in taking part in this particular interview/research etc.

BACKGROUND INFORMATION (provides context for remainder of data) – Interviewees' experience of UC (symptoms, diagnosis, treatment, impact)

Brief discussion of period leading to diagnosis (e.g. when, symptoms, impact, presentation, initial treatment, knowledge of UC, reaction to diagnosis)

Brief discussion of experience post diagnosis (e.g. experience of flare and remission, treatment, impact, adaptations)

PERSPECTIVES ON TRIAL RECRUITMENT, PARTICIPATION AND PROCEDURES

1. What are your reasons/motivations for taking part in STOP-COLITIS?
    - How long did it take you to decide to take part?
    - Did you make the decision on your own or did you ask other people's opinion to help you decide? If so, who? And why?
2. Patients' understanding of STOP-COLITIS
    - In your own words, what do you think the aims of STOP-COLITIS are?
    - What were you told about it?
3. Patients' understanding/expectations of FMT
    - Were you aware of FMT before being introduced to STOP-COLITIS?

For peer review only

- What is your understanding of FMT? NG? Colon?
- What were you told about FMT before consenting to the trial?
- How do you feel about receiving someone else's stool sample?
- How do you think it compares with other treatments (medical treatment)?
- Do you know what the two groups (NG/Colon) entail?
- Do you have any preferences as to which group you would like to be randomised to?
- Have you talked with other people about the trial or FMT? Have you told your partner/close family about what FMT is?
- How would you react to being randomised the NG group? The Colonoscopy group?
- What do your close family and partner/parents think about FMT?

4. Expectations for trial participation
   - What do you expect from taking part in STOP-COLITIS?
5. Experience of information and consent received from medical team
   - Type of information received
   - Who provided the information?
   - Was it useful?
   - Enough time to digest it? To think about the study?
   - What other information would patients have liked (if any)?
6. Potential barriers/facilitators to patients' participation in STOP-COLITIS
   - What might influence patients to participate?
   - Why might patients decide not to take part in STOP-COLITIS?
   - What could be done to encourage them taking part in STOP-COLITIS?
7. Experience of different stages of trial
   - How would you describe your experience of consenting to STOP-COLITIS?
   - Who took consent?
   - Did you have enough time to think about it before consenting?
   - Do you know what "random allocation" is in STOP-COLITIS and what it involves? Who did the randomisation?
   - Do you know what the follow-up process entails in STOP-COLITIS? Questionnaires/Blood tests/stool samples etc…?
8. Outcomes
   - What should we be comparing/measuring in these questionnaires etc…?
   - What is important to you (in terms of symptoms, treatment, QoL, etc…)

For peer review only

9. The main trial, after this pilot trial, will include a placebo group and an intervention group (NG or colon?), hypothetically, Would you have felt differently about STOP-COLITIS if there was a possibility that you might get the placebo rather than the FMT?
10. I have come to the end of my questions, do you have any other issues you wish to talk about?

CLOSING

Tell the participants that they have reached the end of the interview

Check understanding of any outstanding points and give the participant the opportunity to answer ask any further questions.

Check arrangements for scheduling the follow up interview

Remind them about confidentiality and thank them for their time

For peer review only

INTERVIEW SCHEDULE STOP-COLITIS T2 (12 weeks) - PATIENTS

INTRODUCTION

Thank the participant for agreeing to take part.

Ensure that the consent form has been signed. Keep a copy for the site file and give a copy back to the participant.

Statement on confidentiality, right to withdraw consent, recording of the interview.

Review the purpose of the study in general.

Emphasize the value of their views and opinions – there are no right or wrong answers.

Ask if the participant has any questions before starting the interview.

HOW PATIENTS FEEL AFTER 12 WEEKS MONTHS

1. How have you been in general since the last time I saw you?
2. What symptoms have you experienced over the last 3 months? (any changes since initial interview?)
3. Have you had any flare ups since initial interview? Types/Severity/Frequency of symptoms? Treatment?
4. Has anything changed since the last time I saw you?

PILOT TRIAL PROCESSES AND PROCEDURES

1. How did you feel about being randomised to NG/Colon?
   - Were you hoping to be randomised to NG/Colon?
2. What was your experience of FMT (NG)?
   - Colonoscopy?
   - Medication prior to/after FMT infusion?
   - Intubation?
   - Attending the CRF daily?
   - Did it match your expectations?
   - Do you wish you had been randomised to the other group? Why? Why not?
3. What was your experience of FMT (Colon) and recovery?
   - Colonoscopy?
   - Medication prior to/after FMT dose?
   - Endoscopy?
   - Enema?

For peer review only

- Attending the CRF weekly?

4. What was your experience of the clinical reviews/assessments after the intervention?
    - What did you think of the QoL questionnaires you had to complete?
    - What did you think of IBD diaries? Did you complete them? How did you find it?
    - What did you think of the stool kit? Was it easy to use?
    - Is there anything that could have been done differently as far as these follow-up assessments are concerned?
5. Overall, have your initial expectations of taking part in the trial been met? Have these expectations changed?
6. What do you think are the barriers and facilitators to patients' participation in STOP-COLITIS?
7. Is there anything that you think we should do differently?

CLOSING

Tell the participants that they have reached the end of the interview.

Check understanding of any outstanding points and give the participant the opportunity to answer ask any further questions.

Remind them about confidentiality and thank them for their time.

For peer review only

INTERVIEW SCHEDULE STOP-COLITIS – WITHDRAWAL PATIENTS

INTRODUCTION

Thank the participant for agreeing to take part

Ensure that the consent form has been signed. Keep a copy for the site file and give a copy back to the participant

Statement on confidentiality, right to withdraw consent, recording of the interview

Review the purpose of the study in general

Emphasize the value of their views and opinions – there are no right or wrong answers

Ask if the participant have any questions before starting the interview

BACKGROUND/ICE BREAKER

What are your main reasons for agreeing to talk to me (qual researcher)?

HOW PATIENTS FEEL AFTER 3 MONTHS

5. How have you been in general since the last time I saw you?
6. What symptoms have you experienced over the last 3 months? (any changes since initial interview?)
7. Have you had any flare ups since initial interview? Types/Severity/Frequency of symptoms? Treatment?
8. Has anything changed since the last time I saw you?

PILOT TRIAL PARTICIPATION AND PROCEDURES

8. How did you feel about being randomised to NG/Colon?
    - Were you hoping to be randomised to NG/Colon?
9. What was your experience of FMT (NG)?
    - Colonoscopy?
    - Medication prior to/after FMT infusion?
    - Intubation?
    - Attending the CRF daily?
    - Did it match your expectations?
    - Do you wish you had been randomised to the other group? Why? Why not?
10. What was your experience of FMT (Colon)?
    - Colonoscopy?

For peer review only

- Medication prior to/after FMT dose?
- Endoscopy?
- Enema?
- Attending the CRF weekly?

11. What was your experience of the clinical reviews/assessments after the intervention? *(if applicable)*
    - What did you think of the QoL questionnaires you had to complete?
    - What did you think of IBD diaries? Did you complete them? How did you find it?
    - What did you think of the stool kit? Was it easy to use?
    - What do you we should have done differently as far as these follow-up assessments are concerned?
12. Overall, have your initial expectations of taking part in the trial been met? Have these expectations changed?

REASONS FOR WITHDRAWING FROM STOP-COLITIS

13. What were your main reasons for withdrawing from the trial?
    - Did you take the decision on your own? Did you ask for other people's advice? If so, who?
14. What else would make patients decide to withdraw from the study?
15. What could be done to prevent patients from withdrawing from the study?
16. What do you think we should do differently overall?

CLOSING

Tell the participants that they have reached the end of the interview

Check understanding of any outstanding points and give the participant the opportunity to answer ask any further questions.

Remind them about confidentiality and thank them for their time

For peer review only

INTERVIEW SCHEDULE STOP-COLITIS – PATIENT DECLINERS

INTRODUCTION

Thank the participant for agreeing to take part

Ensure that the consent form has been signed. Keep a copy for the site file and give a copy back to the participant

Statement on confidentiality, right to withdraw consent, recording of the interview

Review the purpose of the study in general

Emphasize the value of their views and opinions – there are no right or wrong answers

Ask if the participant have any questions before starting the interview

BACKGROUND/ICEBREAKER

Background, interviewee details and icebreaker e.g. explore interviewee background, whether they have family, etc…

BACKGROUND INFORMATION (provides context for remainder of data) – INTERVIEWEES' EXPERIENCE TO DATE WITH UC (symptoms, diagnosis, treatment, impact)

Brief discussion of period leading to diagnosis (e.g. when, symptoms, impact, presentation, initial treatment, knowledge of UC, reaction to diagnosis)

Brief discussion of experience post diagnosis (e.g. experience of flare and remission, treatment, impact, adaptations)

PERSPECTIVES ON TRIAL RECRUITMENT AND PARTICIPATION

11. Patients' understanding of STOP-COLITIS
    - In your own words, what do you think the aims of STOP-COLITIS are?
    - Do you know what "random allocation" is in STOP-COLITIS and what it involves?
12. Patients' understanding/expectations of FMT
    - Were you aware of FMT before being introduced to STOP-COLITIS?
    - What is your understanding of FMT? NG? Colon?
    - Do you know what the two groups (NG/Colon) entail?
13. Experience of information received from medical team
    - Type of information received

For peer review only

- Who provided the information?
- Was it useful?
- Would patients have liked other information? If so, what information?

REASONS FOR DECLINING TO PARTICIPATE

14. What are your reasons/motivations for not taking part in STOP-COLITIS?
    - How long did it take you to decide not to take part?
    - Did you make the decision on your own or did you ask other people's opinion to help you decide? If so, who? And why?
15. Potential barriers/facilitators to STOP-COLITIS
    - What might influence patients to participate?
    - What else might make patients decide not to take part in STOP-COLITIS?
    - What could be done to encourage them taking part in STOP-COLITIS?
16. What do you think we could do differently overall?
17. Do you have any other issues you wish to talk about?

CLOSING

Tell the participants that they have reached the end of the interview

Check understanding of any outstanding points and give the participant the opportunity to answer ask any further questions.

Remind them about confidentiality and thank them for their time

For peer review only

Reporting checklist for protocol of a clinical trial.

Based on the SPIRIT guidelines.

		Reporting Item	Page Number
Title	#1	Descriptive title identifying the study design, population, interventions, and, if applicable, trial acronym	1
Trial registration	#2a	Trial identifier and registry name. If not yet registered, name of intended registry	3
Trial registration: data set	#2b	All items from the World Health Organization Trial Registration Data Set	3
Protocol version	#3	Date and version identifier	3
Funding	#4	Sources and types of financial, material, and other support	3
Roles and responsibilities: contributorship	#5a	Names, affiliations, and roles of protocol contributors	18
Roles and responsibilities: sponsor contact information	#5b	Name and contact information for the trial sponsor	19
Roles and responsibilities:	#5c	Role of study sponsor and funders, if any, in study design; collection, management, analysis, and interpretation of	17

For peer review only

sponsor and funder		data; writing of the report; and the decision to submit the report for publication, including whether they will have ultimate authority over any of these activities	
Roles and responsibilities: committees	#5d	Composition, roles, and responsibilities of the coordinating centre, steering committee, endpoint adjudication committee, data management team, and other individuals or groups overseeing the trial, if applicable (see Item 21a for data monitoring committee)	18
Background and rationale	#6a	Description of research question and justification for undertaking the trial, including summary of relevant studies (published and unpublished) examining benefits and harms for each intervention	4
Background and rationale: choice of comparators	#6b	Explanation for choice of comparators	4
Objectives	#7	Specific objectives or hypotheses	6
Trial design	#8	Description of trial design including type of trial (eg, parallel group, crossover, factorial, single group), allocation ratio, and framework (eg, superiority, equivalence, non-inferiority, exploratory)	6
Study setting	#9	Description of study settings (eg, community clinic, academic hospital) and list of countries where data will be collected. Reference to where list of study sites can be obtained	6

For peer review only

Eligibility criteria	#10	Inclusion and exclusion criteria for participants. If applicable, eligibility criteria for study centres and individuals who will perform the interventions (eg, surgeons, psychotherapists)	9
Interventions: description	#11a	Interventions for each group with sufficient detail to allow replication, including how and when they will be administered	11
Interventions: modifications	#11b	Criteria for discontinuing or modifying allocated interventions for a given trial participant (eg, drug dose change in response to harms, participant request, or improving / worsening disease)	11
Interventions: adherance	#11c	Strategies to improve adherence to intervention protocols, and any procedures for monitoring adherence (eg, drug tablet return; laboratory tests)	n/a
Interventions: concomitant care	#11d	Relevant concomitant care and interventions that are permitted or prohibited during the trial	9
Outcomes	#12	Primary, secondary, and other outcomes, including the specific measurement variable (eg, systolic blood pressure), analysis metric (eg, change from baseline, final value, time to event), method of aggregation (eg, median, proportion), and time point for each outcome. Explanation of the clinical relevance of chosen efficacy and harm outcomes is strongly recommended	7
Participant timeline	#13	Time schedule of enrolment, interventions (including any	12

For peer review only

		run-ins and washouts), assessments, and visits for participants. A schematic diagram is highly recommended (see Figure)	
Sample size	#14	Estimated number of participants needed to achieve study objectives and how it was determined, including clinical and statistical assumptions supporting any sample size calculations	6
Recruitment	#15	Strategies for achieving adequate participant enrolment to reach target sample size	n/a
Allocation: sequence generation	#16a	Method of generating the allocation sequence (eg, computer-generated random numbers), and list of any factors for stratification. To reduce predictability of a random sequence, details of any planned restriction (eg, blocking) should be provided in a separate document that is unavailable to those who enrol participants or assign interventions	10
Allocation concealment mechanism	#16b	Mechanism of implementing the allocation sequence (eg, central telephone; sequentially numbered, opaque, sealed envelopes), describing any steps to conceal the sequence until interventions are assigned	10
Allocation: implementation	#16c	Who will generate the allocation sequence, who will enrol participants, and who will assign participants to interventions	10
Blinding (masking)	#17a	Who will be blinded after assignment to interventions (eg,	n/a

For peer review only

		trial participants, care providers, outcome assessors, data analysts), and how	
Blinding (masking): emergency unblinding	#17b	If blinded, circumstances under which unblinding is permissible, and procedure for revealing a participant's allocated intervention during the trial	n/a
Data collection plan	#18a	Plans for assessment and collection of outcome, baseline, and other trial data, including any related processes to promote data quality (eg, duplicate measurements, training of assessors) and a description of study instruments (eg, questionnaires, laboratory tests) along with their reliability and validity, if known. Reference to where data collection forms can be found, if not in the protocol	15
Data collection plan: retention	#18b	Plans to promote participant retention and complete follow-up, including list of any outcome data to be collected for participants who discontinue or deviate from intervention protocols	15
Data management	#19	Plans for data entry, coding, security, and storage, including any related processes to promote data quality (eg, double data entry; range checks for data values). Reference to where details of data management procedures can be found, if not in the protocol	18
Statistics: outcomes	#20a	Statistical methods for analysing primary and secondary outcomes. Reference to where other details of the statistical analysis plan can be found, if not in the protocol	15

For peer review only

Statistics: additional analyses	#20b	Methods for any additional analyses (eg, subgroup and adjusted analyses)	n/a
Statistics: analysis population and missing data	#20c	Definition of analysis population relating to protocol non-adherence (eg, as randomised analysis), and any statistical methods to handle missing data (eg, multiple imputation)	n/a
Data monitoring: formal committee	#21a	Composition of data monitoring committee (DMC); summary of its role and reporting structure; statement of whether it is independent from the sponsor and competing interests; and reference to where further details about its charter can be found, if not in the protocol. Alternatively, an explanation of why a DMC is not needed	16
Data monitoring: interim analysis	#21b	Description of any interim analyses and stopping guidelines, including who will have access to these interim results and make the final decision to terminate the trial	16
Harms	#22	Plans for collecting, assessing, reporting, and managing solicited and spontaneously reported adverse events and other unintended effects of trial interventions or trial conduct	15
Auditing	#23	Frequency and procedures for auditing trial conduct, if any, and whether the process will be independent from investigators and the sponsor	15
Research ethics approval	#24	Plans for seeking research ethics committee / institutional review board (REC / IRB) approval	17
Protocol	#25	Plans for communicating important protocol modifications	17

For peer review only

amendments		(eg, changes to eligibility criteria, outcomes, analyses) to relevant parties (eg, investigators, REC / IRBs, trial participants, trial registries, journals, regulators)	
Consent or assent	#26a	Who will obtain informed consent or assent from potential trial participants or authorised surrogates, and how (see Item 32)	9
Consent or assent: ancillary studies	#26b	Additional consent provisions for collection and use of participant data and biological specimens in ancillary studies, if applicable	n/a
Confidentiality	#27	How personal information about potential and enrolled participants will be collected, shared, and maintained in order to protect confidentiality before, during, and after the trial	18
Declaration of interests	#28	Financial and other competing interests for principal investigators for the overall trial and each study site	19
Data access	#29	Statement of who will have access to the final trial dataset, and disclosure of contractual agreements that limit such access for investigators	18
Ancillary and post trial care	#30	Provisions, if any, for ancillary and post-trial care, and for compensation to those who suffer harm from trial participation	n/a
Dissemination policy: trial results	#31a	Plans for investigators and sponsor to communicate trial results to participants, healthcare professionals, the public,	18

For peer review only

		and other relevant groups (eg, via publication, reporting in results databases, or other data sharing arrangements), including any publication restrictions	
Dissemination policy: authorship	#31b	Authorship eligibility guidelines and any intended use of professional writers	18
Dissemination policy: reproducible research	#31c	Plans, if any, for granting public access to the full protocol, participant-level dataset, and statistical code	18
Informed consent materials	#32	Model consent form and other related documentation given to participants and authorised surrogates	n/a
Biological specimens	#33	Plans for collection, laboratory evaluation, and storage of biological specimens for genetic or molecular analysis in the current trial and for future use in ancillary studies, if applicable	14

The SPIRIT checklist is distributed under the terms of the Creative Commons Attribution License CC-BY-ND 3.0. This checklist was completed on 25. March 2019 using https://www.goodreports.org/, a tool made by the EQUATOR Network in collaboration with Penelope.ai

For peer review only
